# Revisiting the *Plasmodium falciparum* druggable genome using predicted structures and data mining

**DOI:** 10.1038/s44386-025-00006-5

**Published:** 2025-03-04

**Authors:** Karla P. Godinez-Macias, Daisy Chen, J. Lincoln Wallis, Miles G. Siegel, Anna Adam, Selina Bopp, Krypton Carolino, Lauren B. Coulson, Greg Durst, Vandana Thathy, Lisl Esherick, Madeline A. Farringer, Erika L. Flannery, Barbara Forte, Tiqing Liu, Luma Godoy Magalhaes, Anil K. Gupta, Eva S. Istvan, Tiantian Jiang, Krittikorn Kumpornsin, Karen Lobb, Kyle J. McLean, Igor M. R. Moura, John Okombo, N. Connor Payne, Andrew Plater, Srinivasa P. S. Rao, Jair L. Siqueira-Neto, Bente A. Somsen, Robert L. Summers, Rumin Zhang, Michael K. Gilson, Francisco-Javier Gamo, Brice Campo, Beatriz Baragaña, James Duffy, Ian H. Gilbert, Amanda K. Lukens, Koen J. Dechering, Jacquin C. Niles, Case W. McNamara, Xiu Cheng, Lyn-Marie Birkholtz, Alfred W. Bronkhorst, David A. Fidock, Dyann F. Wirth, Daniel E. Goldberg, Marcus C. S. Lee, Elizabeth A. Winzeler

**Affiliations:** 1 Department of Pediatrics, University of California, San Diego, La Jolla, CA USA; 2 Panorama Global, 2101 4th Ave, Ste 2100, Seattle, WA USA; 3 Lgenia, Inc., 412 S Maple St, Fortville, IN USA; 4 MMV Medicines for Malaria Venture, 1215, Geneva, Switzerland; 5 Department of Immunology and Infectious Diseases, Harvard T.H. Chan School of Public Health, Boston, MA USA; 6 Holistic Drug Discovery and Development (H3D) Centre, Institute of Infectious Disease and Molecular Medicine, University of Cape Town, Cape Town, South Africa; 7 Department of Microbiology and Immunology, Columbia University Irving Medical Center, New York, NY USA; 8 Center for Malaria Therapeutics and Antimicrobial Resistance, Division of Infectious Diseases, Department of Medicine, Columbia University Irving Medical Center, New York, NY USA; 9 Department of Biological Engineering, Massachusetts Institute of Technology, Cambridge, MA USA; 10 Division of Infectious Diseases, Boston Children’s Hospital, Boston, MA USA; 11 Global Health, Biomedical Research, Novartis, Emeryville, CA USA; 12 Drug Discovery Unit, Division of Biological Chemistry and Drug Discovery, School of Life Science, University of Dundee, Dundee, UK; 13 Skaggs School of Pharmacy and Pharmaceutical Sciences, University of California, San Diego, La Jolla, CA USA; 14 Calibr-Skaggs Institute for Innovative Medicines, a division of The Scripps Research Institute, La Jolla, CA USA; 15 Division of Infectious Diseases, Washington University School of Medicine, Saint Louis, MO USA; 16 São Carlos Institute of Physics, University of São Paulo, São Carlos, São Paulo, Brazil; 17 Center for Systems Biology, Massachusetts General Hospital, Boston, MA USA; 18 TropIQ Health Sciences, Nijmegen, The Netherlands; 19 Infectious Disease and Microbiome Program, Broad Institute, Cambridge, MA USA; 20 Global Health Drug Discovery Institute, Beijing, China; 21 Global Health Medicines R&D, GlaxoSmithKline, Tres Cantos, Madrid, Spain; 22 Department of Biochemistry, Genetics & Microbiology, Institute for Sustainable Malaria Control, University of Pretoria, Private Bag X20, Hatfield, Pretoria, South Africa; 23 Department of Molecular Microbiology, Washington University School of Medicine, Saint Louis, MO USA; 24 Division of Biological Chemistry and Drug Discovery, Wellcome Centre for Anti-Infectives Research, University of Dundee, Dundee, UK

**Keywords:** Target identification, Target validation

## Abstract

Identification of novel drug targets is a key component of modern drug discovery. While antimalarial targets are often identified through the mechanism of action studies on phenotypically derived inhibitors, this method tends to be time- and resource-consuming. The discoverable target space is also constrained by existing compound libraries and phenotypic assay conditions. Leveraging recent advances in protein structure prediction, we systematically assessed the *Plasmodium falciparum* genome and identified 867 candidate protein targets with evidence of small-molecule binding and blood-stage essentiality. Of these, 540 proteins showed strong essentiality evidence and lack inhibitors that have progressed to clinical trials. Expert review and rubric-based scoring of this subset based on additional criteria such as selectivity, structural information, and assay developability yielded 27 high-priority antimalarial target candidates. This study also provides a genome-wide data resource for *P. falciparum* and implements a generalizable framework for systematically evaluating and prioritizing novel pathogenic disease targets.

## Introduction

Over the past decade, phenotypic screening has gained popularity since large, diverse compound libraries can be tested in a high-throughput fashion without requiring a priori knowledge of targets or mechanisms of action^1^. After triage, a subset of screening hits is typically subjected to target identification, facilitating target-based medicinal chemistry programs. In malaria parasites, this approach has revealed new targets^1,2^, including P-type cation translocating ATPase 4 (ATP4)^3^, acetyl-CoA synthetase (ACAS)^4^, and several aminoacyl-tRNA synthetases^5–8^. Nonetheless, novel targets are urgently needed to develop drugs that differ from existing antimalarials in mechanism of action and resistance liability.

Compound-dependent target discovery faces several limitations. Limited chemical matter and follow-up on only the most potent phenotypic hits pose restrictions on the targeted biology space. As a result, studies tend to reidentify targets such as *Pf*DHODH and *Pf*ATP4^1^. In addition, target identification is an arduous process, especially when targets lack known ortholog inhibitors in other species. Alternatively, in silico approaches can identify proteins amenable to target-based drug discovery^1,9^. For malaria parasites, key characteristics of a candidate target are its “druggability” (i.e., ability to be modulated by a drug-like ligand), and its essentiality to parasite survival in the lifecycle stage of interest. Although the druggable genome of the deadliest human malaria species, *Plasmodium falciparum*, has been explored via in silico methods^10,11^, these studies have relied on homology to known targets and drug–gene interactions to predict druggability^11,12^. Homology-based protein modeling tools like SWISS-MODEL^13^ and molecular docking tools like AutoDock Vina^14^, while useful for virtual screening^1^, are less appropriate for assessing proteome-wide druggability. With the advent of artificial intelligence models for predicting protein structure, such as AlphaFold^15^ and ESMFold^16^, along with ligand-binding prediction tools like AlphaFill^17^, we are now able to comprehensively assess the whole genome for essential proteins with evidence of small-molecule binding. This approach may identify drug targets overlooked by compound-dependent discovery efforts.

Leveraging the collective expertise of the Malaria Drug Accelerator (MalDA)^2^ consortium—a partnership between academia and industry aiming to accelerate antimalarial drug discovery—we systematically identified and ranked a list of “druggable target” candidates from the entire *P. falciparum* genome that could progress into drug discovery. The list was determined by identifying genes with evidence of both protein binding to small molecules^17–19^ and essentiality in the parasite asexual blood stage (ABS)^20–22^. Their viability as drug target candidates was further assessed based on common characteristics of known drug targets using available literature and data^1,23–26^. In addition to highlighting promising blood-stage antimalarial targets, we provide an in-depth annotation resource that can facilitate future target validation and lead optimization efforts. By predicting ligand-target interactions from AlphaFill^17^, BindingDB^18^, and BRENDA^19^, we uncovered some small-molecule tool compounds for further studies or as starting points for structure activity relationship (SAR) design. The framework used in this study to integrate and evaluate druggability information may be applied to genome-wide in silico target discovery for other pathogenic organisms and diseases.

## Results

### Defining 1660 *P. falciparum* genes with evidence of small-molecule binding

**Fig. 1:**
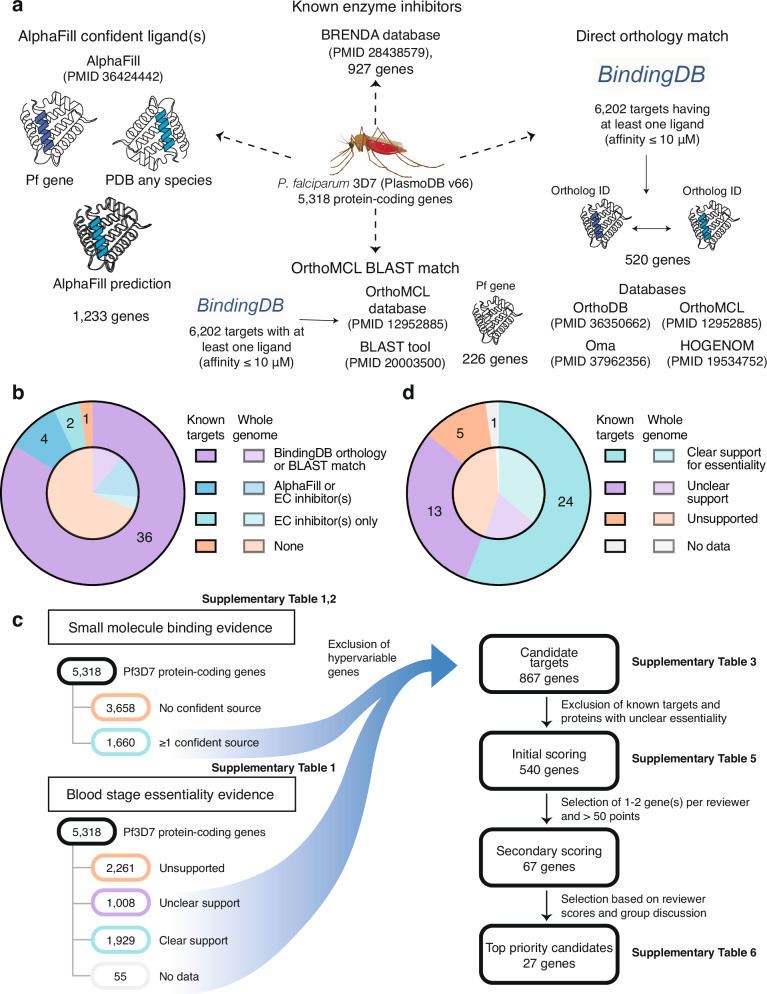
Identification of *P. falciparum* protein-coding genes that are potentially druggable (*n* = 1660) and genes that have evidence of blood-stage essentiality (*n* = 2992). **a** Identification of potentially druggable genes. Diagram illustrating the four methods used to identify genes with evidence of small-molecule binding. Out of the 5318 protein-coding 3D7 *falciparum* genes, 226 were homologous to at least one of the 6202 validated drug targets in BindingDB based on BLAST, while 520 genes were found to be orthologous based on the phylogenomic databases OrthoMCL, OrthoDB, OMA, or HOGENOM. We found 1233 genes with confident AlphaFill hit transplant(s), and 927 *falciparum* genes were mapped to EC numbers with inhibitors in the BRENDA database. Mosquito graphic was generated with BioRender (https://BioRender.com/s17b944). **b** Binding evidence for validated targets vs. all genes. Distribution of binding evidence for 43 known targets^1^ (outer pie) compared to the distribution across all 5275 *P. falciparum* 3D7 genes (inner pie). **c** Workflow for the identification of 867 candidate targets and subsequent prioritization. Identification and filtering process to define *P. falciparum* candidate drug targets. The intersection of 1660 genes with evidence of small-molecule binding and 2992 genes with essentiality support yielded 867 candidates, after excluding hypervariable regions. Subsequent candidate prioritization resulted in 540 candidates subjected to initial scoring, 67 of which underwent a second round of scoring, culminating in 27 top-ranking targets that were discussed by a panel of experts. **d** Essentiality classifications for validated targets vs. all genes. Distribution of essentiality classifications for 43 known targets^1^ (outer pie) compared to the distribution for 5275 3D7 genes (inner pie). Essentiality classifications are described in “Methods”.

From 5318 protein-coding genes in the *P. falciparum* 3D7 genome (PlasmoDB^27^ release 66), we identified a set of proteins that are “ligandable” and, therefore, potentially druggable. Proteins were evaluated by integrating data from predictions of ligands based on similarity to existing co-crystallized protein structures using AlphaFill^17^, orthology or sequence similarity to proteins with experimentally determined protein–ligand-binding affinities in BindingDB^18^, and curated enzyme-inhibitor interactions in BRENDA^19^ (Fig. 1a and Supplementary Tables 1 and 2).

Of the 5099 *P. falciparum* 3D7 genes with an AlphaFold protein model, 2771 had at least one AlphaFill “hit”, i.e., sufficient local sequence homology to a protein in the PDB-REDO databank^28^ associated with ligand(s), referred to as potential “transplants”. We restricted our attention to 1233 proteins that had at least one confident AlphaFill transplant with global RMSD (root-mean-square-deviation, a measure of structural similarity) < 10 and local RMSD < 4, thresholds informed by empirical observation. Precipitants commonly used in protein crystallization and small ligands (< 10 atoms) were ignored, as they are unlikely to be drug-like (“Methods”). To broaden druggability evidence for *P. falciparum* enzymes and overcome the fact that many *P. falciparum* proteins are not orthologous to crystallized proteins, we incorporated information on inhibitors linked to EC (Enzyme Commission) number classes in the BRENDA database^19^. This yielded 321 additional proteins lacking confident AlphaFill predictions (Supplementary Table 1). We further augmented our ligandable set using 6202 targets (UniProt IDs) from BindingDB^18^, a curated database of experimentally determined protein–ligand-binding affinities. Of 5318 *P. falciparum* proteins, 581 were orthologous to at least one of the 6202 BindingDB targets based on OrthoMCL^23^, OMA^25^, HOGENOM^29^, or OrthoDB^24^ phylogenomic databases, or based on BLAST^30^ hits (*E* value < 1) to the OrthoMCL full protein database (Supplementary Table 2).

Altogether, we found 1660 unique proteins with at least one source of small-molecule binding evidence. Many (*n* = 817) were identified by only one source (Supplementary Fig. 1), demonstrating the importance of multiple evidence sources to reduce false negatives. The set may include a few false positives; one possible example is the apical membrane antigen 1 (AMA1, PF3D7_1133400), an essential vaccine candidate lacking evidence of classical druggability. In crystallography studies^31^, Akter et al. used a peptide probe with the spin label MTSL, also known as (1-Oxyl-2,2,5,5-tetramethylpyrroline-3-methyl)-methanethiosulfonate, which was identified as an AlphaFill hit and is of similar size to small-molecule inhibitors^31^. More sophisticated approaches than molecular weight filtering may be needed to reduce false positives, highlighting the need for expert review, as described below.

To assess our approach, we examined a set of 43 known *P. falciparum* antimalarial targets that have some level of clinical, in vivo, or in vitro validation^1^. We found that, except for NCR1 (PF3D7_0107500), all were supported by at least one source of binding evidence (Fig. 1b). Twenty-six of the 43 validated targets were well-known enzyme targets such as DHFR-TS (PF3D7_0417200) and DHODH (PF3D7_0603300) that had AlphaFill hit(s), ortholog(s) in BindingDB and known enzyme class inhibitors from BRENDA. In five cases (eEF2, elongation factor 2; CPSF3, cleavage and polyadenylation specificity factor subunit 3; FNT, formate-nitrite transporter FNT; PF3D7_1038900, a monoacylglycerol lipase-like esterase; and MQO, malate:quinone oxidoreductase), a single source of binding evidence rescued the validated target.

### Defining 1929 *P. falciparum* genes with evidence of blood-stage essentiality

To assess which of the 5318 *P. falciparum* protein-coding genes are required for asexual parasite growth, we incorporated essentiality data for *P. falciparum* and the rodent malaria species *P. berghei*, reasoning that orthologous genes could provide additional information on essentiality (Supplementary Fig. 2). We focused on the asexual blood stage due to its role in the manifestation of clinical symptoms as well as completeness of available essentiality screens. In the *P. falciparum* screen by Zhang et al.^20^, 3271 proteins were labeled essential for in vitro ABS growth based on genome-wide transposon mutagenesis. Among 2383 *falciparum* orthologs of *berghei* genes tested with gene disruption vectors in the PlasmoGEM dataset^21^, 1145 were essential in the ABS, and the RMgmDB dataset^22^ indicated a change in phenotype upon gene modification for 1319 of 1609 *P. falciparum* genes whose *berghei* orthologs were tested^21,22^.

Reasoning that ambiguous essentiality data should not preclude proteins from consideration as targets, we created a categorization scheme for the strength of essentiality evidence (Supplementary Fig. 3). Categories were defined as “clear support”, “unclear support”, “unsupported”, or “no data” for essentiality in the asexual blood stage. For “clear support”, all available evidence sources must confidently label the protein as essential; if either Zhang et al. or PlasmoGEM confidently labeled the protein as nonessential, it was considered “unsupported”, while “unclear support” describes all other proteins with data from at least one source.

In total, 1929 *P. falciparum* proteins were classified as having clear essentiality support, 1008 with unclear support, 2326 with support for non-essentiality, and 55 with no data (Fig. 1c). Surprisingly, this classification scheme categorized five of the 43 validated targets from Siqueira-Neto et al.^1^ (MQO, PDEdelta, PNP, PF3D7_1038900, and PMX) under unclear support (Fig. 1d). In all but one case, either the *P. falciparum* or *berghei* datasets suggest the protein is essential while at least one source is contradictory. The exception was PDEδ (PF3D7_1470500, cGMP-specific 3’,4’-cyclic phosphodiesterase δ), which regulates erythrocyte deformability^32^ and is not a blood-stage target but rather the target of tadalafil in mature gametocyte stages. While these results show that available data are sometimes inconsistent and can only partially inform *Plasmodium* gene essentiality, by combining multiple evidence sources, we increased our confidence that proteins categorized as “clear support” are essential and thus potential antimalarial targets.

### 867 *P. falciparum* proteins have evidence of binding and blood-stage essentiality

**Fig. 2:**
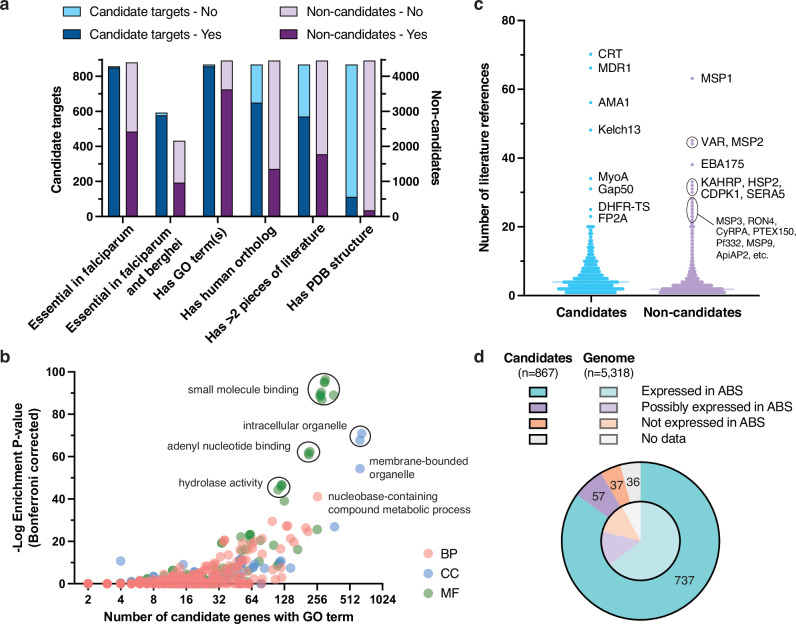
Characteristics of 867 candidate targets compared to 4451 non-candidate genes. **a** Characteristics of candidate targets vs. non-candidates. Comparison of characteristics between the 867 candidates (blue) and 4451 non-candidates (purple). Numbers of candidate targets versus non-candidates labeled essential only in the *P. falciparum* essentiality screen and not the *P. berghei* datasets (850 vs. 2421); labeled essential in both *P. falciparum and berghei* datasets (577 vs. 967); having at least one GO term (857 vs. 3624); having human ortholog(s) (650 vs. 1356); having > 2 associated literature references (570 vs. 1775); or having PDB structures (112 vs. 174) are shown. **b** Gene ontology (GO) term enrichment analysis for the 867 candidates. GO term enrichment analysis for the 867 candidate targets compared to all 5318 protein-coding genes in the *P. falciparum* 3D7 genome using GOATOOLS^80^. Terms with ontology tree depth > 2 (*n* = 939) are displayed based on the number of candidates having the GO term (*X* axis) versus −log_10_ Bonferroni corrected enrichment *P* value, with a maximum uncorrected *P* value of 0.05 (*Y* axis, Fisher’s exact test). Points corresponding to GO terms are colored by ontology type: red for biological process (BP), blue for cellular component (CC), and green for molecular function (MF). Highly enriched terms or groups of terms are labeled with shared descriptors. **c** Scientific literature references for candidates vs. non-candidates. Distributions of a number of unique scientific publications associated with the 867 candidate targets (blue) vs. 4451 non-candidate genes (purple). Median lines are shown for both groups, and the most highly referenced genes are labeled. **d** Gene expression in the asexual blood stage (ABS) compared to all protein-coding genes. Distribution of classifications for gene expression evidence in ABS (ring, trophozoite, or schizont) according to Le Roch et al.^38^. Candidate targets with clear evidence of ABS expression (737, teal), unclear evidence (57, purple), no expression (37, orange) and no data (36, gray) are shown in the outer pie, in contrast to the distribution among all *P. falciparum* protein-coding genes in the inner pie.

To define an initial list of candidate targets, we took the intersection of the 1660 proteins with small-molecule binding evidence and the 2992 proteins not categorized as “unsupported” (Fig. 1c). This yielded 867 candidate targets after filtering 19 genes in hypervariable noncore regions (Supplementary Table 3). Noncore genes, encompassing *var*, *rifin*, and *stevor* multigene families and other genes in highly recombinogenic subtelomeres^33,34^, were not considered as their variability and redundancy make them poor targets even where deemed essential (e.g., PF3D7_0101600, a rifin with mutagenesis index score of 0.199^20^). The 867 candidate targets were distributed throughout the genome with no apparent propensity for specific chromosomes (Supplementary Fig. 4). Most (*n* = 651) of the 867 candidate targets were supported by AlphaFill binding evidence, 336 were orthologs of or had BLAST matches to validated targets in BindingDB, and 457 were supported by BRENDA enzymatic data. Among 857 candidate targets present in the Zhang et al. *P. falciparum* dataset, 850 were labeled as essential, in contrast to 2421 of 4396 non-candidate proteins (Fig. 2a).

Attractively, 577 of the 867 candidate targets were confidently essential in both *P. falciparum* parasites and the PlasmoGEM (*n* = 452) or RMgmDB (*n* = 162) *P. berghei* datasets (Fig. 2a). This suggests potential as therapeutic targets for multiple *Plasmodium* species, which is important given that antimalarial drugs will need to act against *P. vivax* as well. Among these 577 candidates, we observed known targets (*n* = 7) with validated clinical inhibitors, like eEF2 and PI4K (phosphatidylinositol 4-kinase), and clinically unexplored targets such as BDP1 (PF3D7_1033700, bromodomain protein 1). A small number of candidate proteins (*n* = 14) appeared to be confidently essential in *P. falciparum* but not *P. berghei*, including two acyl-CoA synthetases (PF3D7_0301000, PF3D7_0525100) and two serine/threonine FIKK kinases (PF3D7_0301200, PF3D7_0902400).

### Building an annotation resource using scientific evidence to prioritize candidate targets

To evaluate the 867 candidate targets, we compiled additional annotations for all *P. falciparum* protein-coding genes (Supplementary Fig. 5). We included information on genomic features and genetic variation (PlasmoDB^27^, NCBI^35^, and MalariaGEN^36^), protein features and structures (Protein Data Bank^26^ (PDB) and PlasmoDB), expression across the parasite lifecycle stages (Malaria Cell Atlas^37^ and Le Roch et al.^38^), literature references (NCBI and PubMed), and similarity to human orthologs (Fig. 2a and Supplementary Table 3) (see “Methods” for more details). We reasoned that in addition to druggability evidence, these annotations would allow us to prioritize proteins that merit further structural/functional characterization and target-based screening. The compiled data are accessible via a web resource at pftargetbrowser.org and are summarized in Supplementary Table 3.

Only 286 of the 5318 *P. falciparum* proteins have an experimentally determined structure in PDB database. Of these, 112 were in our list of 867 candidate targets, indicating prior characterization of many candidate targets and highlighting those amenable to structure-based drug design (Fig. 2a). Examples of candidate crystal structures include ferredoxin-NADP reductase (FNR)^39^ and aspartate carbamoyltransferase (ATCase) in complex with a recently discovered small-molecule allosteric inhibitor^40^. We also observed that 2006 protein-coding genes have human orthologs based on OrthoMCL. To estimate the structural similarity of *P. falciparum* proteins to their human counterparts, we performed pairwise comparisons of their AlphaFold models using TM-align^41^. This allowed us to identify the most similar human ortholog for 1972 *P. falciparum* proteins (AlphaFold structures were not available in 34 cases). On average, orthologs showed 33% sequence identity for local alignments that were, on average, 244 amino acids long (Supplementary Fig. 6). A human ortholog was not reported for 217 candidates, which may include promising targets involved in parasite-specific essential biology.

To characterize the biological functions of proteins in the candidate list, we performed Gene Ontology (GO) term enrichment analysis (Fig. 2b). Across the 867 candidates, 857 had at least one associated GO term. The most highly enriched terms (tree depth > 2) were related to small-molecule binding, in particular nucleotide binding (*n* = 299, *P* = 8.2 × 10^−96^, Bonferroni corrected to reduce false positives). The cellular component term “intracellular organelle” was also highly enriched (*n* = 659, *P* = 1.2 × 10^−71^). Closer inspection revealed that these 659 candidates have greater proportions of genes associated with the nucleus (*n* = 371, *P* = 1.3 × 10^−27^), endoplasmic reticulum (*n* = 54, *P* = 9.5 × 10^−7^), food vacuole (*n* = 50, *P* = 6.2 × 10^−12^), and other intracellular organelles compared to all protein-coding genes. Other overrepresented GO terms among candidate targets include ATP binding (*n* = 213, *P* = 2.3 × 10^−62^), pyrophosphatase activity (*n* = 119, *P* = 1.4 × 10^−46^), and more. These results highlight differences in cellular function and localization between proteins predicted to be essential and ligandable and those that are not.

To facilitate candidate target evaluation, we gathered existing evidence by querying literature repositories using PlasmoDB and Entrez gene identifiers (Fig. 2c). Through this approach, we rescued evidence predating the standardization of *Plasmodium* gene nomenclature; for example, four references for *pfhsp101* (PF3D7_1116800) were recovered. Overall, we found literature references for 4956 genes, with a median of four references per gene among candidate targets and two references per gene among non-candidates (Fig. 2c). Unsurprisingly, well-studied genes such as the multidrug resistance genes *pfcrt* (70 references) and *pfmdr1* (66 references), and vaccine targets such as *pfmsp1* (63 references) and *pfama1* (56 references), had the most references.

Finally, to identify candidates with multistage activity, we examined gene expression evidence across the parasite lifecycle using the Le Roch et al. microarray dataset^38^. This dataset is particularly valuable as it includes probabilities of detection above background. As expected, 85% (*n* = 737) of the 867 candidate targets were strongly supported by expression in at least one ABS substage, contrary to 64.5% of all *P. falciparum* genes (Fig. 2d). Of these 737 candidates, 577 also showed strong evidence of expression in the sexual (gametocyte) or mosquito (sporozoite) stages. The remaining 130 candidates were either not measured (*n* = 36), had unclear expression (*n* = 57), or were clearly not expressed across ABS substages (*n* = 37). Around half of these 37 candidate genes also appeared to be minimally expressed according to ABS scRNA-seq data from the Malaria Cell Atlas study^37^, while the other half either contradicted Malaria Cell Atlas expression levels or had dubious evidence of essentiality. In the latter case, many were small proteins (around 100 amino acids long), which have a lower probability of being detected with RNA-seq or tiling microarrays. It is possible that some proteins such as RPUSP (RNA pseudouridylate synthase) and YTH1 (YTH domain-containing protein) are essential despite low expression levels, which could be advantageous for an antimalarial target^42^.

While this work focuses on blood-stage targets for which essentiality and expression data are the most complete, we observed 196 *P. falciparum* orthologs of *P. berghei* genes showing evidence of essentiality in the liver stage^43^ but not in the ABS stage. Of these orthologs, 104 have binding evidence, suggesting their potential as liver stage-specific prophylactic targets.

### Scoring 540 novel candidate targets with strong evidence of essentiality

We next sought to narrow down the candidate targets to those that have strong evidence of essentiality and are relatively novel (limited prior characterization, especially as an antimalarial target). Starting from 587 candidate targets classified as “clear support” for ABS essentiality, we filtered well-known antimalarial targets, such as DHODH and DHFR-TS, validated targets, and target classes currently being pursued by MalDA or others, such as aminoacyl-tRNA synthetases^2^. This resulted in 540 understudied (novel) candidate targets with binding evidence suggesting they are likely to disrupt parasite growth and survival upon perturbation (Fig. 1c).

**Fig. 3:**
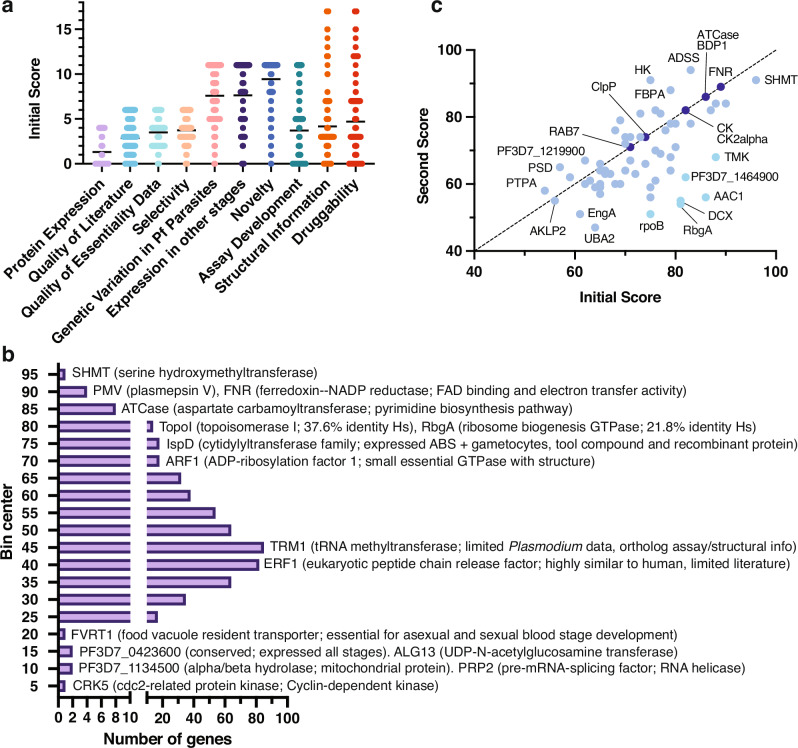
Rubric-based scoring of 540 candidate targets with strong evidence of essentiality. **a** Gene scores across rubric categories. Plot showing first score distributions for 540 candidate targets with strong evidence of blood-stage essentiality across the ten categories of the scoring rubric (“Methods”, Supplementary Table 4). The average value per category is shown for each category. **b** Frequency distribution of first total scores. Histogram showing the frequency (*X* axis) of first total scores (*Y* axis) for the 540 scored candidates. Bin center was determined and plotted with Prism v.9.5. Examples of candidates falling in select total score bins are shown, including gene product description and notable characteristics when applicable. **c** Comparison of first and second scores for the top 67 scored candidates subject to secondary review. Identity line is marked by a dashed black line. Dark blue circles denote equal score in both rounds; light blue circles represent score differences of 1–19 points; and cyan circles represent a score difference of at least 20 points.

Taking advantage of the data compendium, we created a rubric (Supplementary Table 4, “Methods”) to manually score the 540 candidate targets based on their potential for antimalarial target-based drug discovery (Fig. 3a and Supplementary Table 5). Each target was scored on ten weighted categories, totaling to 100 points. The rubric considered the quantity and quality of compiled evidence, the readiness of functional or binding assay development, evidence of druggability and novelty across scientific literature; points were deducted for weak, missing, or contradictory evidence. Some target candidates were deprioritized by reviewers if concerning characteristics regarding their feasibility were observed. For example, reviewers deprioritized CK2α and FKBP35 due to lack of effect on asexual growth from conditional knockout studies^44,45^.

Under this rubric, scores for the 540 novel candidate targets ranged from 6 to 96 points, with an average score of 48.64 (Supplementary Table 5). Candidates with prior characterization tended to receive high scores, while lower scores (≤ 45) were assigned in the absence of recombinant protein expression, biochemical assays, and structural or druggability information. Among 255 low-scoring candidates were subunits of protein complexes, challenging enzyme classes like GTPases, and unsuccessful pre-clinical targets in other organisms. For example, RRP45 (PF3D7_1364500), an RNA exosome complex component, scored 36 points due to a lack of recombinant protein production, lack of a biochemical assay and limited protein structure and tool compound information. Nevertheless, targets with limited prior work scored high in novelty according to our metric (Supplementary Table 5).

Our scoring also revealed attractive high-scoring candidates. One example is TopoI (PF3D7_0510500, topoisomerase I; 80 points), an enzyme involved in DNA replication, transcription, and repair (Fig. 3b). A bacterial TopoI inhibitor^46^ is known, suggesting that the *Plasmodium* enzyme could be selectively targeted. Although our attention was drawn to high-scoring genes, those with lower scores could still be potential drug targets. Such candidates, including the NAD kinase PF3D7_0913300 and proteins that lack human orthologs but are conserved within natural parasite populations like PF3D7_1446800 (heme detoxification protein), will require substantial additional research to confirm their viability as antimalarial targets.

### Secondary scoring of 67 high-ranking candidate targets

Although candidate targets were scored with a predefined rubric, scores were manually determined and could thus vary among reviewers. For example, a reviewer may give a higher score if there is an enzymatic assay specifically for the enzyme under review, whereas another reviewer could give the same score if an enzymatic assay is available for the enzyme class. Therefore, to increase confidence in the scores, we conducted a second round of scoring using the same rubric (Supplementary Table 4), for 67 high-ranking candidates (Fig. 3c and Supplementary Table 5). These 67 candidates were chosen by selecting up to two of the highest-scored proteins recommended by each of the initial reviewers with a minimum first score of 50.

Secondary scoring for the 67 candidates averaged 69.22 points, slightly lower than the first round (73.55 points). Six candidates showed a difference of more than 20 points (Fig. 3c). One example, ribosome biogenesis GTPase A (RbgA), decreased from 81 to 54 points, as the second reviewer placed greater emphasis on the lack of a tool compound and the fact that recombinant protein was only expressed in bacteria. On the other hand, seven genes received the same score from independent reviewers, including ATCase and FNR (Fig. 3c), supporting the rubric’s utility in assessing candidate targets.

### In-depth consideration of 27 prioritized candidates reveals targets poised for drug discovery

From the 67 high-ranking candidate targets with secondary scores, 27 were selected for in-depth consideration by a panel of MalDA experts by once again selecting the top 1–2 candidates recommended by each secondary reviewer. Assessments of target-based drug discovery resources, follow-up strategies, and enablement challenges for the 27 prioritized targets are summarized in Supplementary Table 6. Among these targets, two groups of apicoplast targets were highlighted by different reviewers: caseinolytic protease ATPases (ClpQ, ClpS, ClpY, ClpP, ClpB1) that play important roles in protein homeostasis, and methylerythritol phosphate enzymes (IspD, IspE, IspF) involved in the isoprenoid biosynthesis pathway. We also observed that seven of these 27 prioritized targets lack a human ortholog (Supplementary Table 6), suggesting their potential as highly selective targets.

**Fig. 4:**
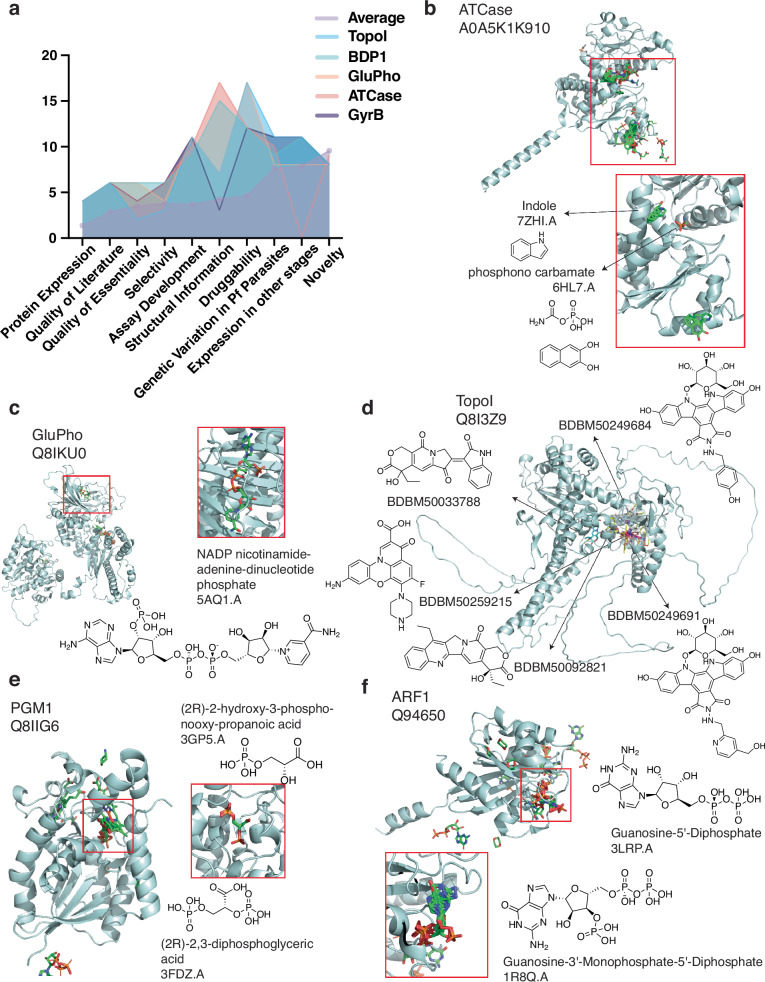
Selection of five high-scoring targets and examples of two understudied but promising candidates. **a** Scoring distribution across categories for top five candidate targets. Individual scores for the top five candidate targets across rubric categories, compared with the average scores across all 540 scored candidates. The average score is highlighted by light purple circles, and top five candidates are shown in blue (TopoI), green (BDP1), orange (GluPho), salmon (ATCase) and dark purple (GyrB). **b**, **c** AlphaFill models for advanced candidate targets. Predicted AlphaFill models for *Pf*ATCase (**b**) and *Pf*GluPho (**c**) are shown. Red rectangles highlight the region where transplant hits were found, with a zoomed-in inset of hit transplant structure having the highest percentage of identity. **d** TopoI model. TopoI (PF3D7_0510500) model was constructed using UniProt ID Q8I3Z9 and ligand hits (Supplementary Table 2). For simplicity, five ligands (BDBM-50249684, −50033788, −50259215, −50249691, and −50092821) associated to the UniProt ID were randomly selected from BindingDB hits. Ligands were docked onto the model using openbabel 3.1.1^81^ and smina 2020.12.10^82^. The model was visualized using PyMol version 2.5.5^83^. **e**, **f** AlphaFill models for understudied but promising candidate targets. Predicted AlphaFill models for *Pf*PGM1 (**e**) and *Pf*ARF1 (**f**) candidate targets. Red rectangles highlight the region of some transplant hits, and a zoomed-in inset including hit transplant structure with the highest percentage of identity is shown.

This exercise also highlighted five attractive targets: ATCase, TopoI, GyrB (DNA gyrase subunit B), GluPho, and BDP1 (Fig. 4a and Supplementary Table 5). These five targets showed the least concerns for progressing in drug discovery efforts according to our rubric, with all but BDP1 having previously demonstrated small-molecule inhibitors^47–50^. Below, we describe ATCase, GluPho, and TopoI, proteins with ongoing work to investigate their potential as antimalarial targets.

ATCase (aspartate transcarbamoylase; Fig. 4b) is a 43.3 kDa protein catalyzing the second step in *Plasmodium*’s de novo pyrimidine synthesis pathway, forming a homo-trimer with three active sites^50^. This pathway is clinically essential since parasites lack a pyrimidine-import pathway, reflected by inhibitors targeting *Plasmodium* DHODH, a downstream enzyme^50^. A truncated version of ATC has been successfully cloned and expressed, and *Pf*ATCase has been crystallized as an apo structure and with a bound allosteric inhibitor^40,51,52^. This enzyme can be measured with phosphate- and carbamoyl aspartate-based assays^52^. Selectivity is achievable as *Hs*ATCase inhibitors, such as PALA analogs, T-state inhibitors, and allosteric inhibitors are not active against *Pf*ATCase^50^. *Plasmodium* inhibitors are known, including Torin2, an ATP-competitive inhibitor with micromolar potency (IC_50_ = 67.7 µM)^53^, and the ligand 2,3-naphthalenediol with medium potency (IC_50_ = 5.5 µM)^50^. Additional SAR or screening of new libraries are needed to identify more suitable inhibitors with drug-like features and tight binding potential.

GluPho (glucose-6-phosphate dehydrogenase-6-phosphogluconolactonase) is another attractive target (Fig. 4c). This bifunctional enzyme catalyzes the first two steps in the pentose phosphate pathway which serves as the major source of NADPH in *Plasmodium*, critical for maintaining parasite redox equilibrium in infected red blood cells^47,54^. Several selective GluPho inhibitors have been identified through target-based screens for *P. falciparum* (e.g., ML276, IC_50_ = 0.89 µM^55^; SBI–0797750, IC_50_ = 0.007 µM^56^; ML304, IC_50_ = 0.19 µM^57^) as well as other organisms such as *Saccharomyces cerevisiae* (e.g., the catechin gallate compound CHEMBL408233 IC_50_ = 21.76 µM^58^). Further work on known series and high-throughput screening for additional *Pf*GluPho inhibitors are warranted to address current liabilities.

TopoI (topoisomerase I) (Fig. 4d), a highly conserved and essential nuclear enzyme, is the only type IB topoisomerase among seven *P. falciparum* topoisomerases^49^. Topoisomerases are well-established targets of anticancer and antibacterial drugs, acting as cellular poisons by selectively trapping the enzyme–DNA cleavage complex^59^. Camptothecin, a classic topoisomerase inhibitor, is potent against erythrocytic parasites^60^, and TopoI shows the highest endogenous activity in schizonts based on functional assays measuring relaxation of supercoiled plasmid DNA, suggesting its role in DNA replication during schizogony^61^. Recombinant expression systems, functional assays, and tool compounds, including some with whole-cell anti-parasite activity, are available for *Pf*TopoI, although selectivity remains a challenge^49,60–62^.

### Secondary reviews suggest 29 understudied candidate targets meriting further characterization

In addition to assessing proteins that were previously explored as antimicrobial targets, our scores inform the feasibility of understudied proteins progressing as novel antimalarial targets. Of the 67 candidate targets with secondary reviews, 29 received the maximum novelty score of 11 points (Supplementary Table 5). Although some characterization is available for these candidates, substantial work is needed to confirm their viability as drug targets. PGM1 (Fig. 4e) and ARF1 (Fig. 4f), novel candidate targets with average scores of 73.5 and 71, respectively, are discussed below.

PGM1 (phosphoglycerate mutase) is involved in glycolysis and gluconeogenesis^63^. It is essential in *P. falciparum* and *berghei* parasites^20,22^, expressed in multiple stages^38^, and has the potential for selectivity against the human enzyme due to differences in protein quaternary structure (tetramer for *falciparum* versus dimer for human). Furthermore, conditional knockdown of *Pf*PGM1 resulted in growth arrest^63^, validating its essentiality. Although inhibitors have not been found, several starting points for validation studies (e.g., selectivity and druggability) and tool compound SAR development against *Pf*PGM1 are available.

ADP-ribosylation factor (ARF1) is a GTPase involved in secretory protein trafficking in eukaryotic cells by initiating vesicle formation at the Golgi apparatus. Our analysis indicates that this enzyme is essential in *falciparum* and *berghei* parasites^20,22^, expressed in sporozoite, gametocyte, and asexual blood stages^37,38^, and has multiple confident AlphaFill transplant hits. This enzyme plays an important role in cancer metastasis; substantial work on human ARF1 has identified diverse inhibitors ranging from the octahydronaphthalene derivative AMF-26^64^ to the triterpenoid natural product demethylzeylasteral^65–67^, providing clues on potential therapeutic strategies against malaria parasites. Although ARF1 has several favorable characteristics, i.e., crystal structure and inhibitors effective against both *Plasmodium* and cancer cells^65–67^, computational prediction and experimental validation are needed to identify effective *Plasmodium* inhibitors since a druggability challenge with small GTPases is the displacement of GTP binding.

## Discussion

Here, we present a systematic data compendium of the *Plasmodium* genome focused on druggability potential as well as an updated set of targets that can readily progress into drug discovery programs. To assess druggability evidence, we leveraged the AlphaFill database of predicted ligand “transplants” based on homology of AlphaFold structures to all structures in the PDB-REDO databank, offering a basis for SAR studies. One concern with this approach is that lax criteria for binding and essentiality evidence may cause the list of 867 “potentially druggable” candidate targets to contain false positives. Many AlphaFill-predicted ligand hits were generic non-drug-like molecules such as ATP; more sophisticated filtering of AlphaFill hits based on chemical properties may improve the predictiveness of this strategy. In addition, few crystal structures exist for *Plasmodium* and apicomplexan parasites compared to other organisms, such as mouse or human; therefore, predicted *P. falciparum* transplant hits found with distant orthologs may not be relevant for malaria parasites, as reflected in low “druggability” scores during expert evaluation.

To minimize these issues, at the cost of deprioritizing completely novel *Plasmodium*-specific candidate targets, we focused on proteins with additional sources of binding evidence such as validated inhibitors in other species. Further validation of predicted ligand “transplants” with putative *P. falciparum* protein targets is needed. It may take several months of SAR to improve affinity strength and inhibitory potency. On the other hand, some proteins with entirely novel modes of binding may be absent from the candidate set if they lack a clear binding pocket or predicted ligand, but are in fact ligandable via a cryptic pocket, i.e., one absent in crystal structures but apparent upon binding of the right ligand. Such cryptic pockets may enable targets in protein classes historically considered undruggable, as in the case of the mutant K-Ras inhibitors^68,69^. Molecular dynamics and/or deep learning approaches to binding pocket prediction may rescue potential false negatives^70–75^.

Another limitation is that manual scoring was only performed on 540 candidate targets with strong ABS essentiality evidence, while targets with ambiguous or conflicting evidence were overlooked. Occasionally, an intermediate relative growth rate labeled “slow” by PlasmoGEM prevented the classification of genes as essential, such as for the known target *Pf*ATP4. Nonetheless, although essentiality may not be fully conserved across *Plasmodium* species, the *P. berghei* essentiality datasets help to validate results from the *P. falciparum* mutagenesis screen, which are less reliable for small genes or those with low TTAA density.

The target evaluation rubric in this study favored proteins with prior characterization and assay development. Due to a focus on “low-hanging fruit”, genes fulfilling alternative criteria, such as hitherto unexplored target classes or *Plasmodium*-specific genes of unknown function, were not highlighted by our ranking. Nevertheless, essential genes with confidently predicted binding hit(s) provide an initial hint that may result in novel target classes, though substantial work is needed since they lack key target fulfillment data.

To date, clinically effective antimalarials with known mechanisms have been limited to drugs targeting known druggable proteins, i.e., those with well-defined, hydrophobic pockets that bind small-molecule ligands. Our study therefore focused on classic druggable proteins, which are more likely to yield small molecule inhibitors with favorable drug-like characteristics. However, new approaches targeting “undruggable” proteins have emerged, such as allosteric inhibitors modulating protein-protein interactions, RNA antisense oligonucleotides (RNAi), and PROTAC (proteolysis-targeting chimera) technology^76^. Thus, it is possible that essential *P. falciparum* genes lacking small-molecule binding evidence in our analysis could be targeted through alternative methods. Nevertheless, the process of selecting genes with small-molecule binding evidence and evidence of asexual stage essentiality (or lack thereof), complemented by clear evidence of target assessment and available resources (e.g., protein expression or structural information), resulted in the prioritization of 27 candidates suitable for target-based drug discovery programs.

We believe the list of candidates proposed in this work can serve as a starting point for future phenotypic validation and small-molecule optimization efforts. As discoveries about protein structure and gene function emerge, automated data extraction and integration will be the next step toward a dynamic resource for prioritizing novel antimalarial targets. As exemplified by a similar target ranking for *Mycobacterium tuberculosis*^9^, this target evaluation approach can be applied to other disease-causing organisms. For *P. falciparum* malaria, our data compendium may assist in prioritizing genes for other use cases, such as vaccine development. Furthermore, our data compendium allowed us to compare characteristics between the list of 1929 genes with essentiality evidence and known drug targets, highlighting those with higher chances of progressing into therapeutic stages that could be obscured otherwise. Overall, we believe this project and the associated website will assist the malaria community in redirecting resources and effort towards future high-quality drug targets.

## Methods

### Data acquisition

List of genes and genomic features (GFF) for *Plasmodium falciparum* 3D7 genome (PlasmoDB release 66) was downloaded and protein-coding genes were extracted along with their gene annotations and genomic location. Additional genomic annotations were obtained by querying PlasmoDB to extract UniProt and Entrez ID(s), ortholog group (OrthoMCL), protein features (CDS and protein length, molecular weight, isoelectric point), domain annotations (InterPro, Pfam, and Superfamily), number of transmembrane (TM) domains, and enzyme commission (EC) numbers. Gene function (Gene Ontology components, functions, and processes) was extracted either from PlasmoDB or by querying the InterPro ID with the InterPro2GO mapping tool from EMBL-EBI. Gene essentiality data was obtained for *P. falciparum*^20^ and *P. berghei*^21,22^ parasites mapped to their *falciparum* orthologs using OrthoMCL orthology group IDs. Protein Data Bank (PDB) IDs of crystal structures were obtained by searching either gene symbols, UniProt IDs associated with each gene, or the term “*Plasmodium*”. A report with gene identifier, organism, accession number, method for structure determination and publication information was extracted for the search hits.

### Mapping genes to associated literature publications

A download from the NCBI FTP site was performed for gene2pubmed.gz (version 2024-02-21) containing taxonomy ID, gene ID (Entrez) and PubMed ID information. Gene IDs were mapped to the *P. falciparum* 3D7 annotation set, and corresponding PMIDs were extracted. To include literature references associated with gene symbols, we queried each symbol in PubMed using the Eutils^77^ efetch function from NCBI; additional information for each publication was obtained pragmatically using the same tool, namely title, authors and digital object identifier (DOI). Literature references from gene nomenclature extraction were manually reviewed and filtered for unrelated records (e.g., same name but different meaning across organisms/diseases).

### Determining candidate proteins with evidence of small-molecule binding

BindingDB^18^ (version 2024-01-01) was queried to extract a list of 6202 unique UniProt IDs with at least one ligand having a measured affinity of at least 10 μM. Ligand SMILES were extracted for target hits. These proteins were queried for sequence similarity against the OrthoMCL^23^ (v.6.19) database using BLAST v2.15 blastp function^30^. Orthology of *P. falciparum* 3D7 proteins to any of the 6202 BindingDB proteins was determined based on presence in the same ortholog group according to OrthoMCL, HOGENOM^29^, OMA^25^ or OrthoDB^24^ phylogenomic databases, using the UniProt ID mapping tool (accessed February 2, 2024). Either direct orthology to a BindingDB protein based on at least one phylogenomic database or a BLAST hit with *E* value < 1 was considered as binding evidence based on BindingDB.

Predictions of ligands corresponding to Pf3D7 AlphaFold (v4) models were taken from the AlphaFill databank^17^, which identifies candidate ligands by searching for sequence homologs in PDB^28^ with known ligands and “transplanting” ligands in regions of local structural homology. AlphaFill excludes common crystallization agents such as polyethylene glycol; in order to focus on AlphaFill hits that are more likely to indicate druggability, we further excluded small ligands with less than ten atoms as well as additional salts, solvents, and polymers used for protein crystallization (PDB ligand IDs: 1BO, ACN, ACT, CCN, CIT, CL, DIO, DMS, EOH, FLC, FMT, GBL, HEZ, IPA, JEF, MLA, MLI, MPD, PDO, PEG, PO4, POL, SBT, SIN, SO4, TBU, TLA) listed in McPherson and Gavira^78^. AlphaFill hits having global RMSD < 10 (structural similarity between the protein of interest and its potential homolog) and local RMSD < 4 (structural similarity of the backbone atoms within 6 Å from the transplanted ligand, after local structural alignment) were considered “confident” hits. Any Pf3D7 protein with at least one confident AlphaFill hit (global RMSD < 10 and local RMSD < 4) to a ligand satisfying the exclusion criteria was classified as having binding evidence based on AlphaFill.

Lastly, inhibitors linked to EC number classes were obtained from BRENDA Enzyme Database^19^ (release 2023.1) by querying EC number annotations for Pf3D7 genes, applicable only to enzymes. Additional ligand types were not considered and for genes with incomplete EC number annotations, all EC numbers matching wildcards were considered. Each Pf3D7 gene with at least one BRENDA EC inhibitor, excluding single-atom ions, was classified as having binding evidence based on BRENDA. Classifications of binding evidence based on orthology or sequence homology to a ligandable protein in BindingDB, presence of confident AlphaFill hit(s), and presence of relevant BRENDA EC inhibitor(s) are listed for each Pf3D7 gene in Supplementary Table 1.

### Identification of human orthologs

Homo sapiens genes (GRCh38, release 39) orthologous to Pf3D7 genes were determined based on OrthoMCL ortholog groups. Sequence and structural similarity were evaluated through pairwise comparison of Pf3D7 and human ortholog AlphaFold (v4) structures with TM-align^41^.

### Definition of hypervariable and core genomic regions

Initial definitions of hypervariable and core regions in the *P*. falciparum 3D7 genome from Miles et al.^79^ were adjusted on a gene-by-gene basis to include most *var*, *rifin*, *stevor*, and *Pfmc-2TM* multigene family members within subtelomeric or internal hypervariable regions. Non-nuclear genome genes were classified according to their respective chromosome (apicoplast or mitochondrial). The genome classifications for each Pf3D7 gene are listed in Supplementary Table 1.

### Categorization of gene essentiality evidence

For each gene, evidence for blood-stage essentiality (Zhang et al., PlasmoGEM and RMgmDB) was classified as either confidently essential, confidently nonessential, unclear, or “no data” if unavailable. Conservative thresholds were used to heuristically categorize genes as confidently essential or nonessential. In the case of the Zhang et al. *piggyBac* insertion mutagenesis dataset, which reports the number of transposon insertions in addition to a Mutagenesis Index Score (MIS), genes labeled with the “Non-Mutable in CDS” phenotype were considered confidently essential if 0 insertions were observed, MIS > 0.8, and the phenotype was not noted as “tentative”. Genes labeled as “Mutable in CDS” were considered confidently nonessential if number of insertions ≥ 1, MIS < 0.5, and the phenotype was not noted as “tentative”. Genes measured by the Zhang et al. dataset that did not fulfill either sets of criteria were categorized as having unclear evidence of essentiality. For PlasmoGEM, genes labeled as “Insufficient data” were included in the “no data” category. A more complex classification scheme was used to rescue essential genes with a “Slow” phenotype by accounting for relative growth rate. If more than 10% or 20% of the 95% confidence interval for relative growth rate fell below 0.5 for genes labeled “Essential” or “Slow”, respectively, or the PlasmoGEM confidence score < 3 for genes labeled “Essential”, evidence was considered unclear; otherwise, genes with the “Essential” phenotype or “Slow” phenotype with relative growth rate < 0.5 were categorized as confidently essential. Meanwhile, genes labeled “Slow” with relative growth rate ≥ 0.5 were confidently nonessential if the lower bound on relative growth rate > 0.6, genes labeled “Dispensable” were confidently nonessential if either the lower bound on relative growth rate ≥ 0.5 or confidence > 3, and genes labeled “Fast” (suggesting increased growth rate upon disruption) were unilaterally considered nonessential. Finally, for RMgmDB, when the phenotype was not “nt” (not tested), evidence was categorized as confidently essential if there was a change in phenotype upon gene modification; if no difference was observed, RMgmDB evidence was considered unclear. Information from the three data sources was integrated to determine gene essentiality bins, which were “full” if all available sources suggest the gene is confidently essential, “anti” if at least one source suggests the gene is confidently nonessential, “partial” if all sources of evidence are unclear, and “no data” if the gene was not tested in any of the three datasets.

### Assessment of gene expression by lifecycle stage

To assess gene expression in the asexual blood stage, genes were categorized based on the strength of evidence from the Le Roch et al. microarray dataset^38^, which reports expression and logP values for six ABS substages synchronized using two different methods. Previous *P. falciparum* 3D7 gene IDs were mapped to current IDs using PlasmoDB. Genes were considered expressed in ABS if at least one substage showed expression value ≥ 30 and logP ≤ −1, not expressed in ABS if all substages showed expression < 10 or logP > −0.5. Otherwise, evidence was considered unclear; such genes were labeled “potentially expressed” in ABS. The Malaria Cell Atlas Chromium 10x RNA-seq dataset was also incorporated in the web resource and Supplementary Table 3; among the four stages tested (ring, trophozoite, schizont, and gametocyte), genes were considered expressed if median expression > 0, and evidence of expression was considered unclear if the third quartile of RNA-seq expression across cells > 0.

### Candidate target scoring rubric

Ten categories belonging to each data type collected were defined to provide a total of 100 points. Availability of recombinant or in situ protein expression was scored between 0 (no information) and 4 (available). Quality of literature and quality of gene essentiality were scored between 0 and 6 points, with higher scores for a greater amount of relevant literature or confident essentiality evidence. For selectivity, 0 was given if the *Plasmodium* protein and human ortholog were very similar (though exact similarity percentages varied between reviewers, a 25–83% range was observed) and data suggested selectivity could be an issue; a maximum score of 6 was given if a human ortholog was absent or there were clear differences between small-molecule inhibitors for *Plasmodium* and human enzymes. Evidence of multistage expression received a score of 3 if there was evidence in one stage, up to 11 for three or more stages. Target novelty, conservation among species (genetic variation), and assay development ranged from 0 to 11 depending on prior characterization of the protein, especially as a potential antimalarial target (novelty); extent of conservation (for genetic variation); or availability of binding/functional assays. Structural information scored from 0 to 17 depending on whether crystal structures were available for orthologs in other organisms, *Plasmodium* proteins, or as ligand-bound structures. Lastly, druggability was scored from 0 (no evidence of binding pocket) to 17 points if a tool compound inhibiting *Plasmodium* growth was known. A detailed description of the scoring rubric is found in Supplementary Table 4.

## Supplementary information

Supplementary information

Supplementary table 1

Supplementary table 2

Supplementary table 3

Supplementary table 5

## Data Availability

Data generated and analyzed during the current study are available in the Supplementary material. Collected information on protein-coding genes in the Pf3D7 genome is showcased at http://pftargetbrowser.org and can be downloaded from 10.6084/m9.figshare.27190545.v1.
