# Supplementary material for: Revisiting the Plasmodium falciparum druggable genome using predicted structures and data mining
Source: NPJ Drug Discov. 2025 Mar 4;2:3. doi: 10.1038/s44386-025-00006-5 (PMC11892419; doi:10.1038/s44386-025-00006-5)
Supplement: Supplementary file 1 — Supplementary information [file 44386_2025_6_MOESM1_ESM.docx]

**Supplementary Material**

**for**

**Revisiting the *Plasmodium falciparum* druggable genome using predicted structures and data mining**

Karla P. Godinez-Macias^1,†^, Daisy Chen^1,†^, J. Lincoln Wallis^2^, Miles G. Siegel^3^, Anna Adam^4^, Selina Bopp^5^, Krypton Carolino^1^, Lauren B. Coulson^6^, Greg Durst^3^, Vandana Thathy^7,8^, Lisl Esherick^9^, Madeline A. Farringer^5,10^, Erika L. Flannery^11^, Barbara Forte^12^, Tiqing Liu^13^, Luma Godoy Magalhaes^12^, Anil K. Gupta^14^, Eva S. Istvan^15^, Tiantian Jiang^1^, Krittikorn Kumpornsin^14^, Karen Lobb^3^, Kyle J. McLean^9^, Igor M. R. Moura^7,16^, John Okombo^7,8^, N. Connor Payne^5,17^, Andrew Plater^12^, Srinivasa P. S. Rao^11^, Jair L. Siqueira-Neto^1,13^, Bente A. Somsen^18^, Robert L. Summers^5,19^, Rumin Zhang^20^, Michael K. Gilson^13^, Francisco-Javier Gamo^21^, Brice Campo^4^, Beatriz Baragaña^12^, James Duffy^4^, Ian H. Gilbert^12^, Amanda K. Lukens^5,19^, Koen J. Dechering^18^, Jacquin C. Niles^9^, Case W. McNamara^14^, Xiu Cheng^20^, Lyn-Marie Birkholtz^22^, Alfred W. Bronkhorst^18^, David A. Fidock^7,8^, Dyann F. Wirth^5,19^, Daniel E. Goldberg^15,23^, Marcus C.S. Lee^24^, Elizabeth A. Winzeler^1,13,*^

**Affiliations**

^1^Department of Pediatrics, University of California, San Diego, La Jolla, CA 92093, USA

^2^Panorama Global, 2101 4th Ave, Ste 2100. Seattle, WA 98121, USA

^3^Lgenia, Inc., 412 S Maple St. Fortville, IN 46040, USA

^4^MMV Medicines for Malaria Venture, 1215 Geneva 15, Switzerland

^5^Department of Immunology and Infectious Diseases, Harvard T.H. Chan School of Public Health, Boston, MA 02115, USA

^6^Holistic Drug Discovery and Development (H3D) Centre, Institute of Infectious Disease and Molecular Medicine, University of Cape Town, Cape Town, South Africa

^7^Department of Microbiology and Immunology, Columbia University Irving Medical Center, New York, NY 10032, USA

^8^Center for Malaria Therapeutics and Antimicrobial Resistance, Division of Infectious Diseases, Department of Medicine, Columbia University Irving Medical Center, New York, NY 10032, USA

^9^Department of Biological Engineering, Massachusetts Institute of Technology, Cambridge, MA 02139, USA

^10^Division of Infectious Diseases, Boston Children’s Hospital, Boston, MA 02115, USA

^11^Global Health, Biomedical Research, Novartis, Emeryville, CA, USA

^12^Drug Discovery Unit, Division of Biological Chemistry and Drug Discovery, School of Life Science, University of Dundee, Dundee DD1 5EH, UK

^13^Skaggs School of Pharmacy and Pharmaceutical Sciences, University of California, San Diego, La Jolla, CA 92093, USA

^14^Calibr-Skaggs Institute for Innovative Medicines, a division of The Scripps Research Institute, La Jolla, CA 92037, USA

^15^Division of Infectious Diseases, Washington University School of Medicine, Saint Louis, MO 63130, USA

^16^São Carlos Institute of Physics, University of São Paulo, São Carlos, São Paulo 13563-120, Brazil

^17^Center for Systems Biology, Massachusetts General Hospital, Boston, MA 02114, USA

^18^TropIQ Health Sciences, 6534 AT, Nijmegen, The Netherlands

^19^ Infectious Disease and Microbiome Program, Broad Institute, Cambridge, MA 02142, USA

^20^Global Health Drug Discovery Institute, Beijing, 100192, China

^21^Global Health Medicines R&D, GlaxoSmithKline, Tres Cantos, Madrid 28760, Spain

^22^Department of Biochemistry, Genetics & Microbiology, Institute for Sustainable Malaria Control, University of Pretoria, Private Bag X20, Hatfield, 0028 Pretoria, South Africa

^23^Department of Molecular Microbiology, Washington University School of Medicine, Saint Louis, MO 63130, USA

^24^Division of Biological Chemistry and Drug Discovery, Wellcome Centre for Anti-Infectives Research, University of Dundee, Dundee, DD1 5EH, UK

^†^ Contributed equally

^*^ Corresponding author. Email: ewinzeler@health.ucsd.edu

Table of Contents

[Supplementary Table 1. Small molecule binding and blood stage essentiality evidence for all P. falciparum 3D7 protein-coding genes. 4](#_Toc187323755)

[Supplementary Table 2. P. falciparum ortholog to UniProt protein IDs extracted from BindingDB having validated ligands. 4](#_Toc187323756)

[Supplementary Table 3. List of 867 candidate targets with evidence of small molecule binding and blood stage essentiality. 4](#_Toc187323757)

[Supplementary Table 4. Target evaluation rubric. 6](#_Toc187323758)

[Supplementary Table 5. Ranking scores for 540 selected druggable candidate targets. 6](#_Toc187323759)

[Supplementary Table 6. Description and proposed strategies for top 27 target candidates. 13](#_Toc187323760)

[Supplementary Figure 1. Sources of small molecule binding evidence for Plasmodium falciparum 3D7 protein-coding genes. 14](#_Toc187323761)

[Supplementary Figure 2. Sources of blood stage essentiality evidence for Plasmodium falciparum 3D7 protein-coding genes. 14](#_Toc187323762)

[Supplementary Figure 3. Essentiality categorization determination scheme for 5,318 P. falciparum protein-coding genes for target identification. 15](#_Toc187323763)

[Supplementary Figure 4. Genome location of the 867 candidate targets with binding evidence and strong essentiality evidence. 16](#_Toc187323764)

[Supplementary Figure 5. Data collected for full genome. 17](#_Toc187323765)

[Supplementary Figure 6. Local sequence identity and structural similarity between orthologs for 1,972 P. falciparum genes having a close human ortholog. 18](#_Toc187323766)

[References 19](#_Toc187323767)

Supplementary Table 1. Small molecule binding and blood stage essentiality evidence for all *P. falciparum* 3D7 protein-coding genes.

Attached Excel spreadsheet. Gene symbols and product descriptions were taken from PlasmoDB^1^ v66 assembly. Binding evidence and essentiality evidence classes were determined based on availability and strength of support from different sources (**Methods**), which were used to construct a list of potentially druggable candidate targets. All data used to assess support from different sources are shown, including AlphaFill hits to PDB entries and predicted ligand transplants, exact ortholog or BLAST matches to proteins with known binding affinity in BindingDB^2^, BRENDA^3^ inhibitors linked to EC number annotations, *P. falciparum* piggyBac mutagenesis data from Zhang *et al.* 2018^4^, *P. berghei* gene disruption phenotypes from PlasmoGEM^5^, and *P. berghei* gene modification phenotypes in RMgmDB^6^ (Rodent Malaria genetically modified Parasites Database).

Supplementary Table 2. *P. falciparum* ortholog to UniProt protein IDs extracted from BindingDB having validated ligands.

Attached Excel spreadsheet. UniProt protein IDs extracted from BindingDB^2^, having at least one ligand with affinity of 10 µM or lower. Taxon, description, core/peripheral population and group ID for each UniProt were extracted from UniProt database^7^. Cases for *Plasmodium* ortholog matches using group ID were found for Pf Match - OrthoMCL^8^, Pf Match - HOGENOME^9^, Pf Match - OMA^10^, and Pf Match - OrthoDB^11^. *Plasmodium* genes having an orthology match according to BLAST^12^ (E-value < 1) are indicated in Pf Match - OrthoMCL BLAST. BRENDA^3^ EC Numbers were found by PlasmoDB^1^ Release 66.

Supplementary Table 3. List of 867 candidate targets with evidence of small molecule binding and blood stage essentiality.

Attached Excel spreadsheet. Information collected for 867 candidate target genes. Data is split by category type, arranged in no particular order. Gene details are based on PlasmoDB^1^ v66 assembly. Human orthology was determined using ortholog groups and TM-align^13^ tool. Binding evidence class is indicated according to **Supplementary Table 1**. Gene expression across the parasite life cycle is based on the Le Roch *et al.* microarray dataset^14^ and Malaria Cell Atlas Chromium 10x RNA-seq dataset^15^ (**Methods**). Genetic variation was mapped using the Pf7 dataset^16^.

| **Quality of Literature** | **Quality of Essentiality Data** | **Conservation (Genetic Variation) in Pf Parasites** | **Evidence of Expression in other stages** | **Novelty** | **Recombinant or in situ Protein Expression** | **Assay Development** | **Selectivity** | **Structural Information** | **Druggability** |
| --- | --- | --- | --- | --- | --- | --- | --- | --- | --- |
|  | 0 = no/weak evidence that the target is essential | 0 = highly variable protein or no close orthologs in Plasmodium species. |  | 0 = compound in MalDA development pipeline, clinical candidate, etc. | 0 = no prior evidence of expression | 0 = no precedent for developing an assay for this target class | 0 = Pf protein is very similar to human homolog | 0 = no structural information available | 0 = no evidence of small molecule binding pocket |
| 2 = literature information is minimal, restricted to Pf | 2 = Zhang screen shows target is essential |  | 3 = only expressed in ABS | 3 = limited data in MalDA but is a known/ developed target class in malaria |  | 1 = Binding assay have been developed for target class in any organism | 2 = Pf protein is very similar to human homolog | 3 = distant ortholog structure solved, homology model constructed | 3 = evidence of small molecule binding pocket, no information in other species |
| 3 = literature information is minimal, exists for Pf and another species |  | 5 = moderately variable protein. Clear orthologs in Plasmodium species and isolates | 5 = expressed in 2 life cycle stages (ABS but not mature gametocytes) | 5 = not in MalDA developed but target class developed in malaria |  | 2 = Binding assay have been developed for Plasmodium or closely related ortholog | 3 = Pf protein has some differences to human homolog | 7 = close ortholog structures solved, homology model constructed | 7 = evidence of a small molecule binding pocket, tool compound exists for other specie |
|  | 4 = Zhang and another screen shows target is essential |  | 8 = expressed in ABS and mature gametocytes | 8 = target has not been explored in MalDA and there is limited work external to MalDA |  | 5 = Functional assay developed for this target class in any specie | 4 = Pf protein has clear differences to human homolog; or human homolog is not essential | 12 = apo or holo structure resolved with (>2.3 A); holo structure of close ortholog resolved | 12 = tool compound for malaria exists or a good small molecule inhibitor tool exists in other species |
| 6 = high quality and significant amount of supporting literature on the target for both Pf and other species | 6 = Zhang, berghei, and other screens show clear evidence of essentiality | 11 = highly conserved with few nonsynonymous variants distinguishing parasites isolates (e.g. HSP90, actin) | 11 = expressed in 3+ life cycle stages (including ABS, liver, and gametocytes) | 11 = target has not been explored within MalDA and there is no ongoing work external to MalDA | 4 = prior evidence of protein expression | 11 = Functional assay(s) has been developed for Plasmodium or closely related protein ortholog | 6 = No close ortholog, inhibition of small molecule inhibitor differences between Pf and Hs enzymes | 17 = structure of target with ligand is available at high resolution (<2.3 A) | 17 = tool compounds with drug-like characteristics exists; demonstrates parasite growth inhibition |

Supplementary Table 4. Target evaluation rubric.

Designed rubric for scoring data compendium for each gene target. Ten categories were defined to assess the literature retrieved, druggability and essentiality data, variation in *Plasmodium* parasites, expression across parasite lifecycle, novelty of target (e.g. limited prior work), protein selectivity, expression and structural information, and assay development feasibility. Total of possible points are color coded ranging from red (weak/no evidence) to dark green (desirable evidence). Descriptions for suggested point assignations are included.

Supplementary Table 5. Ranking scores for 540 selected druggable candidate targets.

Attached Excel spreadsheet. Scores provided for 540 candidates across each category. Scores are based on data collected (**Supplementary Table 3**). Scores result in a total of 100 points, both first and second review (top 67 genes, unless gene unreliable). Individual category scores belong to first category. Reasoning for difference between second versus first review is indicated, when provided by the reviewer.

| **Target** | **Background** | **Enablement** | **Challenges** | **Strategy** | **Scores** |
| --- | --- | --- | --- | --- | --- |
| PF3D7_0206700  ADSL (adenylosuccinate lyase) | - Involved in purine salvage, an essential process - Essential in *P. falciparum*^4^ and *berghei*^5^ screens   - Expressed in asexual, oocysts and mosquito sporozoite stages - Human ortholog: ENSG00000239900 | - Recombinant expression of active enzyme^17^ - *P. vivax* structure (AF-E1CEX9-F1-v4) and *falciparum*^18^ exists - Tool compound available^19^ | - No publications for 15 years - May not be hitting target - Need better tool compound - Small polar active sites are challenging | - Investigate why research halted - Evaluation of pathway and look at all the enzymes to understand function - HTS library screen to identify drug-like inhibitor starting points​ | 80, 78 |
| PF3D7_1354500  ADSS (adenylosuccinate synthetase) | - Catalyzes the Mg2+ dependent formation of adenylosuccinate from IMP and aspartate - Involved in purine salvage pathway. - Essential in *P. falciparum*^4^ - Human orthologs: ENSG00000035687, ENSG00000185100 | - Structure for aspartate analog^18^ - The conversion of IMP to succinyl-AMP and enzyme kinetics were analyzed^20^ - Compounds for bacterial ADSS known^21,22^ | - No inhibitor studies in *falciparum* cultures, *in vitro* enzyme assays only - There may be chemistry liabilities - Hypoxanthine might be issue for readout/bypass | - Confirm *falciparum* essentiality and likely confirm for other species - Perform ABS assay - Identify *falciparum* tool compound with analog or library screening | 83, 94 |
| PF3D7_1344800  ATCase  (aspartate carbamoyltransferase) | - Catalyzes the second step in *falciparum* pyrimidine biosynthesis​^23^ - Other drugs targets the pathway^24^ - Allosteric ligand identified^25^ - Human ortholog: N/A | - Truncated functional enzyme has been cloned and expressed​^26^ - Multiple assays available^27^ - X-ray structure available with citrate bound in orthosteric site (5ILN) and with allosteric ligand bound​ (6FBA) | - Active site is extremely polar, may not identify drug-like orthosteric inhibitors - Need for human selectivity - Parasite inhibition likely to have a lag phase (like DHODH)​ | - Structure-guided on 2,3-dihydroxynaphthalene to improve druggability - Virtual screening - HTS using small molecule library ​ - Clone and express functional enzyme and assay | 78, 86 |
| PF3D7_1033700  BDP1  (bromodomain protein 1) | - Reader of histone acetylated lysine^28^ - Mediates RBC invasion^29^​ - Protein expressed in *E. coli*^30^ - Essential in *P. falciparum*^4^ and *berghei*^5^ parasites - Human ortholog: N/A | - *Pf*BDP1 expressible​^29^ - Phenotypic ABS active inhibitors^31^ - Apo *Pf*BDP1 structure (7m97)​ and *Pf*BDP4 structure available (5vs7)​ | - New ligands may require extensive HTS screening​ - Availability of additional libraries for screening - Literature compounds must be vetted/validated | - Develop biochemical target assays​ - Find new inhibitors​ - Pharmacophore and docking studies of available libraries | 86, 86 |
| PF3D7_1108400  CK2α  (casein kinase 2, alpha subunit) | - Serine/threonine-selective protein kinase implicated in cellular processes^32^ - Essential for merozoite invasion and gametocytogenesis^32^ - Human orthologs: ENSG00000070770, ENSG00000101266, ENSG00000254598 | - Conditional knockdown of *Pf*CK2α expression^32^ - Prevented transition of stage IV into stage V gametocytes^32^ - Crystallographic structure available^33^ - Tool compounds available^33,34^ | - Challenging to develop a HTS assay - Selectivity may be challenging - cKD shows no effect on ABS growth^32^ (but β regulatory subunit seems to be essential in this stage) | - Evaluate essentiality in ABS, currently unclear - Find potential allosteric sites (not yet explored) - Redesign compounds | 82, 82 |
| PF3D7_0307400  ClpP  (ATP-dependent Clp protease proteolytic subunit) | - Part of ATP-dependent Clp multisubunit complex^35^ - ClpS regulates activity of ClpA/P chaperone/protease itself^35^ - Human ortholog: ENSG00000125656 | - Structure solved for P. *falciparum* (2f6i) - Parasite-active probes identified^36,37^ - Proteomics data available (PlasmoDB) | - Small binding site - Need whole complex to be functional - Not classic biochemical pathway - Interfering with degron recognition drives cell death | - Verify essentiality - Identify inhibitors that disrupt the adaptor function of ClpP - Variants of FP assay could be used for HTS | 77, 74 |
| PF3D7_1320100  ClpS  (ATP-dependent Clp protease adapter protein ClpS) | - Predicted to function as an adaptor protein that delivers N-end rule substrates to the ClpA/P^35^ - Bacterial ClpS recognizes N-terminal degrons and delivers the substrate to ClpA/P^35^ - Expressed in many stages^15^ - Human ortholog: N/A | - Crystal structure of *falciparum* fragment solved^35^ - *E. coli* crystal structure solved (3O2B) - Predicted small molecule binding sites (N-terminal peptide degrons)^35^ - Functional assays available^35^ | - Small binding site based on *E. coli* ClpS^35^ - Need whole complex to be functional - Not classic biochemical pathway - Interfering with degron recognition drives cell death | - Identify inhibitors that disrupt the adaptor function of ClpS - Variants of FP assay could be used for HTS | 77, 69 |
| PF3D7_0907400  ClpY  (ATP-dependent protease ATPase subunit ClpY) | - Multimeric system consisting of a small barrel of two stacked hexameric rings of ClpQ protease with the active site facing inside the barrel^38^ - Essential by *falciparum* parasites^4^ - Human ortholog: N/A | - *Pf*ClpY/Q recombinantly expressed individually^39^ - Assays for protease activity and binding available^39^ - Homology model available^40^ - Small molecules reported^40^ | - Recombinant expression of protein complex could be challenging - High through put assay to identify compounds that disrupt ClpY/Q complex needs to be developed | - Identify disruptors of ClpY/Q complex - Gather more information to determine next steps/assays to perform - Artemisinin resistant parasite quiescent state susceptible to mitochondrial drugs | 72, 73 |
| PF3D7_1444800  FBPA  (fructose-bisphosphate aldolase) | - Central enzyme in glycolysis^41^ - Expressed across lifecycle^15^ - Therapeutic target in oncology^41^ - Human orthologs: ENSG00000109107, ENSG00000136872, ENSG00000149925 | - Structural information for *falciparum* available (1A5C, 4TR9, 2EPH) - A novel mode of action is reported^42^ - Compounds that bind to the enzyme active site inhibit aldolase-TRAP interaction, and vice versa^43^ | - Selectivity concerns - Compounds binding other proteins may have effects or vice versa - Requires stoichiometric amounts of inhibitor since it’s not catalytic event | - Modify functional assays for HTS screening - Confirm druggability - Identify where are the inhibitors binding - FRET assay to screen for protein protein interaction - Knockdown evaluation | 79, 88 |
| PF3D7_1247400  FKBP35  (peptidyl-prolyl cis-trans isomerase FKBP35) | - Belongs to the immunophilin family^44^ and regulates many functions^44^ - Essential in *falciparum*^4^ parasites - Human orthologs: ENSG00000004478, ENSG00000096060, ENSG00000119782, ENSG00000198225 | - *Pf*FK506-binding protein with rapamycin available^45^ - *Pf*FK506 and *Pv*FK506 binding domains in complex with D44^46^ - Genetically validated as a target^44^ - Conditional knockdown and knockout^44^ | - FKBP35 requires near maximal knockdown in order to observe a growth phenotype | - Identify additional tool compounds - Test inhibition - Evaluate phenotypic response | 71, 74 |
| PF3D7_0623200  FNR  (ferredoxin--NADP reductase) | - Plant-type ferredoxin-NADP+ reductase, yields reduced ferredoxin for biosynthetic pathways in the apicoplast^47^ - Expressed in trophozoites, rings, gametocytes, and ookinetes^15^ - Human ortholog: N/A | - *Plasmodium* protein has been expressed^47^, and structure is solved ^48^ - Small molecule binding site known^48^ - Tool compound available^48^ | - Spectrophotometric assay is likely not amenable to HTS - Better tool compound may be needed | - Design luciferase assay to study enzymatic activity - SAR to find small molecule inhibitors | 89, 89 |
| PF3D7_1453800  GluPho  (glucose-6-phosphate dehydrogenase-6-phosphogluconolactonase) | - Bifunctional enzyme that catalyzes first two steps of the pentose phosphate pathway^49^ - Source for parasites highly sensitive to oxidative stress^49,50^ - Human ortholog: ENSG00000160211 | - Recombinant expression of *Pf*GluPho, *Pf*G6PD, *Hs*G6PD and *Hs*6PGL^51^ - Ligands are competitive with G6P^52,53^ | - No *Plasmodium* G6PD crystal structure available - Current ligands have liabilities | - Evaluate literature compounds - Potentially perform HTS to find better compounds | 79, 78 |
| PF3D7_0629000  GNA1  (glucosamine 6-phosphate N-acetyltransferase) | - Catalyzes acetylation of GlcN6P to form GlcNAc6P^54^ - Belongs to a family of Gnc5-*N*-acetyltranferases (GNAT)^54^ - Human ortholog: N/A | - Conditional knockout available^55^ - Assay for Crypto enzyme has been developed^54,55^ - Protein binding site identified^55^ - Xray structure of CpGNA1 (6YUG) | - Very polar substrate - No inhibitors identified | - Conditional knockdown needed - Chemical tools need to be identified - Identify lifecycle specificity and rate of kill | 65, 64 |
| PF3D7_1239500  GyrB  (DNA gyrase subunit B) | - Tetrameric protein harbored by apicoplast^56^ - Essential for reducing topological strain while double-stranded DNA is being unwound^57,58^ - Essential in *falciparum*^4^ and *berghei*^5^ parasites - Human orthologs: ENSG00000077097, ENSG00000131747 | - Recombinant protein expressed and biochemical assays available^59,60^ - Fluorescence-mediated biochemical assay available^58^ - Bacterial gyrases and human topoisomerase structures solved^61,62^ | - Structure not available for *Plasmodium* - Fast-killing is unlikely with an apicoplast inhibitor (delayed death effect) - Tool compounds are only weakly active^59^ | - Measure double strand breakage of DNA using fluorescent marker - Identify new compounds using existing (purpurogallin, novobiocin, ciprofloxacin) - On target cellular assays potential (using cKD lines or IPP assay) | 76, 70 |
| PF3D7_1012400  HGPRT  (hypoxanthine-guanine phosphoribosyltransferase) | - Required for purine salvage, catalyzes synthesis of 6-oxopurine mononucleotides - Likely essential in *P.* *falciparum*^4^ parasites, and slow growth in *berghei*^6^ - Expressed across lifecycle^15^ - Human ortholog(s): ENSG00000099256, ENSG00000165704 | - Recombinantly expressed and mechanism characterized^63^ - Crystal structure resolved (3OZF) - Inhibitors against *P.* *falciparum* and *vivax*^64,65^ - IMPDH coupled assay, spectrophotometric shift from NAD+ to NADH measured^63^ | - Selectivity concerns - Protein inhibition potency does not translate to phenotypic potency (good protein inhibitor only)^63-65^ | - Investigate phenotypic response - Find additional inhibitors - Additional/optimize assays - Perform *in vivo* studies | 73, 81 |
| PF3D7_0624000  HK  (hexokinase) | - Involved in glycolysis^66^ - Limited identity to human ortholog in active site^66^ - Human orthologs: ENSG00000156510, ENSG00000156515, ENSG00000159399, ENSG00000160883 | - X-ray crystal structure solved^67^ - ADP-Glo reporter system used to conduct 60K compound screen^66^ - Potential tool compounds^66^ | - Identify good tool compounds - Selectivity and abundance concern | - On-target validation in *Plasmodium* for tool compound (i.e. cKD)​ | 75, 91 |
| PF3D7_0209300  IspF  (2C-methyl-D-erythritol 2,4-cyclodiphosphate synthase) | - Fifth enzyme in the non-mevalonate pathway; isoprenoid biosynthesis precursor^68^ - Forms a homotrimer^68^ - Highly conserved^68^ - Human ortholog: N/A | - Crystal structures available^68^ - Small molecule binding site known^68^ - Fluorescence^69^, and photometric^70^ assays available | - Delayed death/slow rate of kill - No tool compounds available | - Confirm essentiality - Evaluate abundance, functional activity/catalytic efficiency - Computational analysis to identify active site | 76, 82 |
| PF3D7_1446200  LAP  (M17 leucyl aminopeptidase) | - Regulates pool of amino acids, essential for protein synthesis^71^ - Expressed across lifecycle^15^ - Human ortholog: ENSG00000002549 | - Has potent amino acid dipeptide inhibitors^72^ - Recombinant protein available^72^ - Tool compound available^71,72^ and known *T. cruzi* ihibitors^73^ | - Selectivity concerns - If interacting with metal in binding site, could be challenging - Slow killing | - Find long acting inhibitor - Confirm druggability - Cross-specie investigation | 87, 82 |
| PF3D7_0907900  PDF  (peptide deformylase) | - Apicoplast targeted peptide deformylase^74^ - Class has made significant pre-clinical progress^74^ - Expressed in replicating stages and low in female^15^ - Bacterial and oncology inhibitors validated^75^ - Human ortholog: ENSG00000258429 | - Crystal structures known for *Pf*PDF (1RL4) and *Hs*PDF (3G5K) - Structure in apo and peptide-mimic bound states^74,76^ - Expressed in *E. coli*^77^ - Recombinant protein expressed^78^ | - *Pf*PDF may not be the primary target of actinonin^78^ - Limited work published - Active site inhibitors may be difficult to optimize | - SAR around actinonin could make it more active against *Plasmodium* - Generate cKD line | 83, 78 |
| PF3D7_1120100  PGM1  (phosphoglycerate mutase, putative) | - Essential in *falciparum*^4^ and *berghei*^5^ parasites - Differences in *falciparum* (tetramer) vs. human (dimer) structures^79^ - Expressed in multiple stages - Human orthologs: ENSG00000164708, ENSG00000171314 | - cKD validation of essentiality^79^ - *In vitro* and *in vivo* assays available^79^ - Crystal structure available^79^ - Inhibitors described for orthologs^80^ | - Does not have tool compound (in *Plasmodium*) - No selectivity nor druggability information | - Test tool compounds against the *Plasmodium* enzyme and whole cell - SAR to find better tool compound, if needed - Generate conditional knockouts - Computational analysis of active site | 79, 68 |
| PF3D7_1113100  PRL  (protein tyrosine phosphatase) | - Essential signaling enzyme that regulates many cellular processes^81^ - Expressed in ABS, liver and gametocytes^15^ - Human orthologs: ENSG00000112245, ENSG00000184007, ENSG00000184489, ENSG00000275575 | - Recombinant *Pf*PRL is expressed and purified, activity assay available^81,82^ - Inhibitor known to block parasite growth^81^ - *In silico* screens available - Crystal structures available^82^ | - Selectivity concerns - Has human orthologs - Very polar binding site | - Measure activity - Confirm allosteric binding site - Design inhibitors | 70, 74 |
| PF3D7_1144900  RAB6  (ras-related protein Rab-6) | - Crucial role in vesicle transport and membrane trafficking^83^ - Protein transport in medial and trans Golgi cisternae^83^ - Human orthologs: ENSG00000154917, ENSG00000175582 | - Full length and truncated expressed and purified *E. coli* protein available^83^ - Crystal structure available (1D5C) - Tool compounds available for other species | - Shares 67% identity with human Rab6A (4DKX) - Research papers looking at *Pf*RAB6 function are from 2000’s - Not well characterized in *Plasmodium* | - Confirm essentiality in *falciparum* - HTS screen to find possible inhibitors | 76, 61 |
| PF3D7_0410700  RbgA  (ribosome biogenesis GTPase A, putative) | - Essential for biogenesis of the 50S apicoplast large ribosomal subunit​^84^ - Expressed in trophozoite, schizont, and gametocytes^15^ - Human ortholog: ENSG00000148824 | - Recombinant RbgA protein and biochemical assay available^84^ - Several RbgA structures available^85^ | - Available ligand is not drug-like^84^ | - Identify tool compounds - Measure activity | 81, 54 |
| PF3D7_1235600  SHMT  (serine hydroxymethyltransferase) | - Critical enzyme in “one carbon” intermediary metabolism^86^ - Vital for folate recycling^86^ - Essential in *falciparum*^4^ and *berghei*^6^ parasites - Human orthologs: ENSG00000176974, ENSG00000182199, ENSG00000284320 | - Validated druggable pathway in *Plasmodium* (e.g., DHFR inhibitors)^86^ - Recombinant enzyme expressed in *falciparum* and *vivax* (5YG2, 4TMR) - Enzymatic^87^, coupled^86^ and fluorescence^87^ assays available - Tool compound available^88^ | - Antifolate resistance - Pathway or enzymes targeted - Target ID validation not well-established - Metabolic liability concerns - Human selectivity could be challenging | - Target validation - Resistance selections - Structure-guided drug design - Investigate synergy between DHFR and SHMT inhibitors | 96, 91 |
| PF3D7_1251300  TMK  (thymidylate kinase) | - Activation of prodrugs used as antiviral and anticancer drugs^89^ - Essential in *falciparum*^4^ parasites - Expressed in ABS and gametocytes^15^ - Human ortholog: ENSG00000168393 | - Potential drug target against *P. falciparum* but also for other diseases^89^ - Plenty of literature available - Many tool compounds available^90^ - Crystal structures available in various species^90,91^ | - Off-target effects - Selectivity concerns | - Confirm drug targets - Evaluate expression in liver stage (currently unclear) - Generate phenotypic data (less available) | 88, 68 |
| PF3D7_0510500  TopoI  (topoisomerase I) | - Highly conserved enzyme that regulates DNA topology^92^ - Implicated in transcription and DNA replication during the *falciparum* asexual blood-stage^92^ - Human orthologs: ENSG00000184428, ENSG00000198900 | - Assays available^93,94^ - Human ortholog crystal structure known^62^ - Tool compounds exist^95,96^ | - Protein structure and a small molecule inhibitor hit specifically targeting *Pf*Topo1 are not available - Need for selectivity against the human ortholog | - Cloning and expressing functional enzyme and assay - Utilize crystal structure with bound allosteric for virtual screening - Small molecule library HTS for drug-like inhibitors | 80, 71 |
| PF3D7_1460400  UCHL3  (ubiquitin carboxyl-terminal hydrolase isozyme L3) | - Dual-specificity enzyme for ubiquitin and Nedd8^97^ - Likely essential in *falciparum*^4^ parasites - Expressed across lifecycle^15^ - Human orthologs: ENSG00000118939, ENSG00000154277 | - Assay reaction with Ub/Nedd8 probes^97^ - Recombinant protein expressed and structure solved with Ub and UbVME substrates^98^ - Identification of covalent fragment inhibitors for *Pf*UCHL3^99^ | - Transfections of catalytically-dead mutant not successful^98^ | - Confirm essentiality and druggability - Validation with target/cKD - Evaluate in context of druggability | 88, 84 |

# Supplementary Table 6. Description and proposed strategies for top 27 target candidates.

Target knowledge and assessment summary for top 27 target candidates, sorted alphabetically. Each prioritized target contains key information related to target-based drug discovery resources, follow-up strategies, and enablement challenges. Human ortholog is indicated per target. Scores from first and second round are shown for reference.

# Supplementary Figure 1. Sources of small molecule binding evidence for *Plasmodium falciparum* 3D7 protein-coding genes.

Venn diagram partitioning all 5,318 *P. falciparum* 3D7 protein-coding genes according to evidence of protein small molecule binding. Of 1,660 proteins with at least one source of binding evidence, 817 were identified by only one source, while 843 genes were supported by at least two sources. A total of 3,658 proteins did not show binding evidence, and are reported in red.


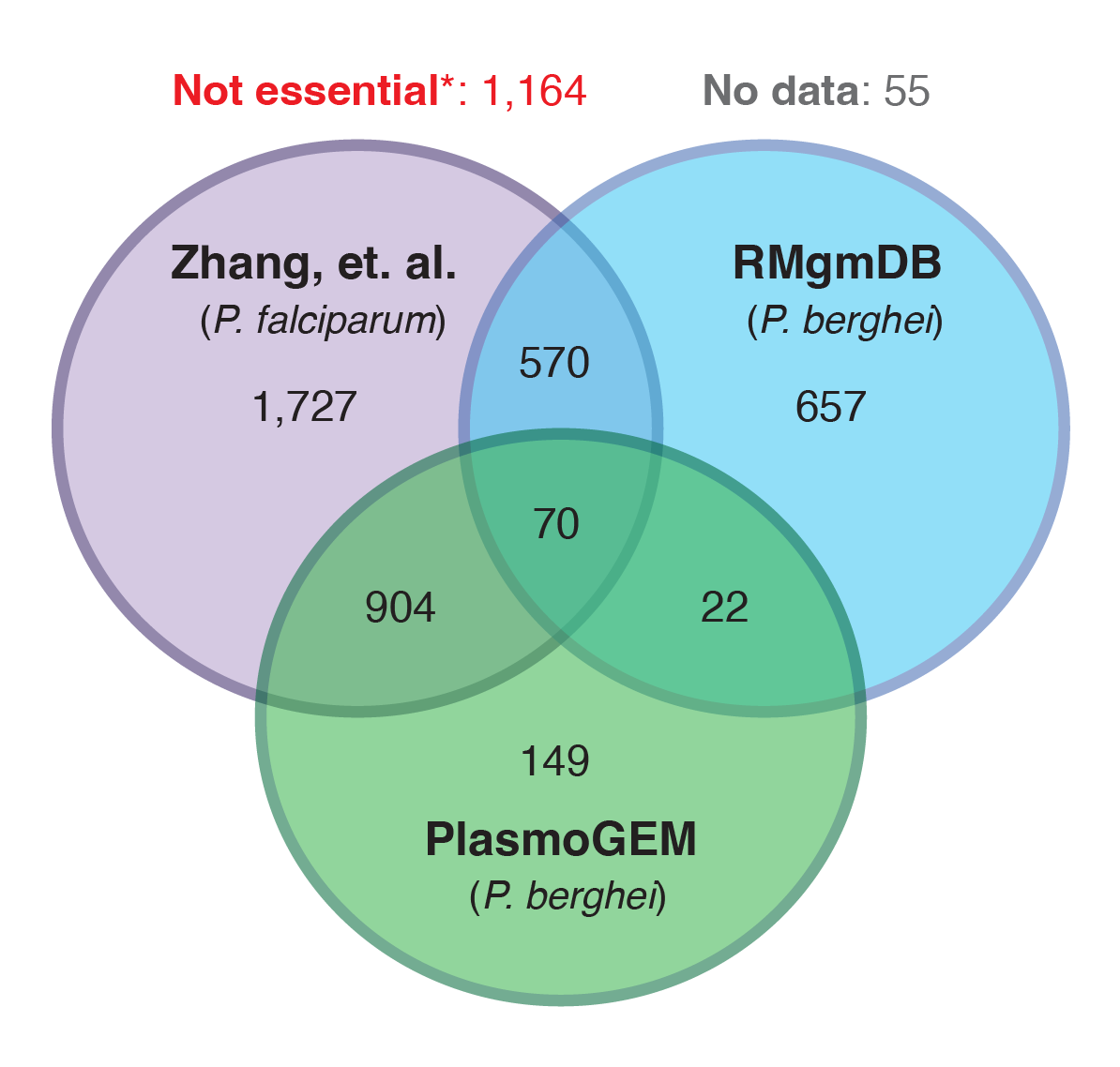


# Supplementary Figure 2. Sources of blood stage essentiality evidence for *Plasmodium falciparum* 3D7 protein-coding genes.

Venn diagram partitioning all 5,318 *P. falciparum* 3D7 protein-coding genes according to essentiality evidence. A total of 3,271 *P. falciparum* genes were essential according to the Zhang *et al.* piggyBac transposon mutagenesis screen^4^, while 1,145 essential *P. falciparum* ortholog genes were found with PlasmoGEM^5^ *P. berghei* dataset, and 1,319 orthologs from RMgmDB^6^. Of 4,099 3D7 genes with essentiality data, 1,566 genes had evidence in *falciparum* and *berghei* parasites. We did not find essentiality data for 55 genes, and 1,164 were defined as not essential.

Supplementary Figure 3. Essentiality categorization determination scheme for 5,318 *P. falciparum* protein-coding genes for target identification.

Criteria to categorize genes in essentiality bins, according to supporting data for essentiality in the asexual blood stage from different datasets^4-6^. Four categories (clear support, unclear support, unsupported, and no data) were defined. Genes under each category based on available data are shown with teal squares for the 1,929 genes having clear essentiality support, purple squares for the 1,008 genes having unclear unsupport, and orange squares for the 2,326 genes categorized as clearly unsupported (e.g., non-essential genes). The remaining 55 genes, in grey, are those lacking essentiality data. Breakdown of genes with support across the datasets is shown.

Supplementary Figure 4. Genome location of the 867 candidate targets with binding evidence and strong essentiality evidence.

Distribution of the 867 candidates across the *P. falciparum* 3D7 genome, according to PlasmoDB^1^ release 66. Five genes located in the apicoplast (*pfrps12*, *pftufa*, *pfclpm*, *pfrpoc1*, *pfrpob*) and two mitochondrial genes (*pfcox1*, *pfcytb*) are not displayed. Colored lines denote the gene position in the chromosome, with blue marks for sense genes and red marks for antisense genes.

**
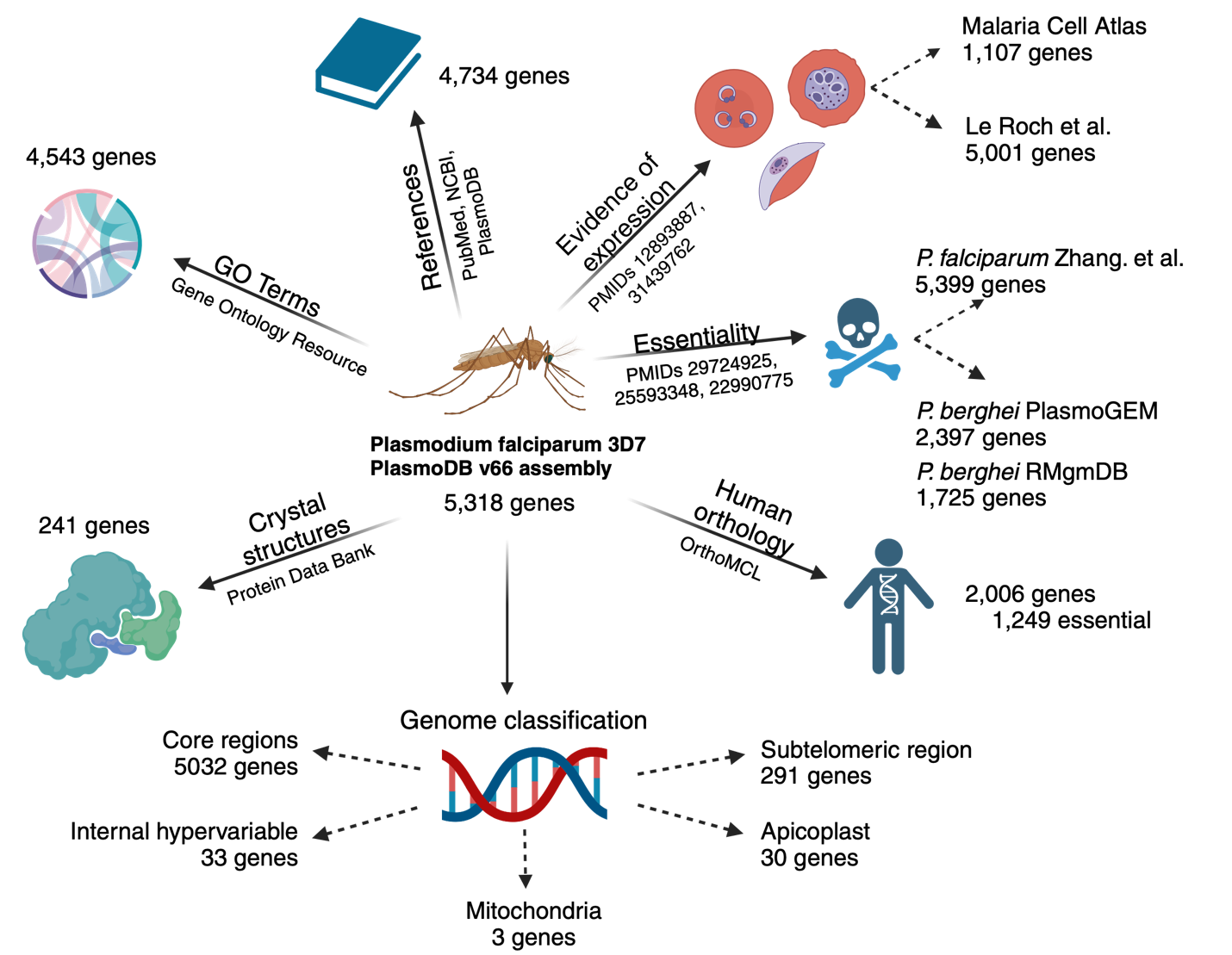
**

Supplementary Figure 5. Data collected for full genome.

Information collected for all 5,318 *P. falciparum* genes from PlasmoDB^1^ release 66. Database resources for collected information are shown^1,8,13,15,100-102^. Total number of genes having at least one record per category is indicated. Out of the 19,900 genes in the human genome, there were 2,006 orthologous *P. falciparum* genes, of which 1,249 were determined to be essential. Genome classification for the 5,318 genes was determined according to **Methods**. Figure was created in BioRender (Godinez, K. (2025) https://BioRender.com/q39w386).

Supplementary Figure 6. Local sequence identity and structural similarity between orthologs for 1,972 *P. falciparum* genes having a close human ortholog. Measures of similarity for 1,972 *falciparum* genes against each gene’s most similar human ortholog. Structural similarity score (0-1) and local alignment sequence identity were calculated with TM-align^13^. Dotted lines mark the average point for sequence identity (Y axis) and structural similarity score (X axis).

#

# References

1 Aurrecoechea, C. *et al.* PlasmoDB: a functional genomic database for malaria parasites. *Nucleic Acids Res* **37**, D539-543 (2009). <https://doi.org:10.1093/nar/gkn814>

2 Liu, T., Lin, Y., Wen, X., Jorissen, R. N. & Gilson, M. K. BindingDB: a web-accessible database of experimentally determined protein-ligand binding affinities. *Nucleic Acids Res* **35**, D198-201 (2007). <https://doi.org:10.1093/nar/gkl999>

3 Schomburg, I. *et al.* The BRENDA enzyme information system: from a database to an expert system. *J Biotechnol* **261**, 194-206 (2017). <https://doi.org:10.1016/j.jbiotec.2017.04.020>

4 Zhang, M. *et al.* Uncovering the essential genes of the human malaria parasite *Plasmodium falciparum* by saturation mutagenesis. *Science* **360**, eaap7847 (2018). <https://doi.org:10.1126/science.aap7847>

5 Schwach, F. *et al.* PlasmoGEM, a database supporting a community resource for large-scale experimental genetics in malaria parasites. *Nucleic Acids Res* **43**, D1176-1182 (2015). <https://doi.org:10.1093/nar/gku1143>

6 Janse, C. J. *et al.* A genotype and phenotype database of genetically modified malaria-parasites. *Trends Parasitol* **27**, 31-39 (2011). <https://doi.org:10.1016/j.pt.2010.06.016>

7 UniProt, C. UniProt: the Universal Protein Knowledgebase in 2025. *Nucleic Acids Res* (2024). <https://doi.org:10.1093/nar/gkae1010>

8 Li, L., Stoeckert, C. J., Jr. & Roos, D. S. OrthoMCL: identification of ortholog groups for eukaryotic genomes. *Genome Res* **13**, 2178-2189 (2003). <https://doi.org:10.1101/gr.1224503>

9 Penel, S. *et al.* Databases of homologous gene families for comparative genomics. *BMC Bioinformatics* **10 Suppl 6**, S3 (2009). <https://doi.org:10.1186/1471-2105-10-S6-S3>

10 Altenhoff, A. M. *et al.* OMA orthology in 2024: improved prokaryote coverage, ancestral and extant GO enrichment, a revamped synteny viewer and more in the OMA Ecosystem. *Nucleic Acids Res* **52**, D513-D521 (2024). <https://doi.org:10.1093/nar/gkad1020>

11 Kuznetsov, D. *et al.* OrthoDB v11: annotation of orthologs in the widest sampling of organismal diversity. *Nucleic Acids Res* **51**, D445-D451 (2023). <https://doi.org:10.1093/nar/gkac998>

12 Camacho, C. *et al.* BLAST+: architecture and applications. *BMC Bioinformatics* **10**, 421 (2009). <https://doi.org:10.1186/1471-2105-10-421>

13 Zhang, Y. & Skolnick, J. TM-align: a protein structure alignment algorithm based on the TM-score. *Nucleic Acids Res* **33**, 2302-2309 (2005). <https://doi.org:10.1093/nar/gki524>

14 Le Roch, K. G. *et al.* Discovery of gene function by expression profiling of the malaria parasite life cycle. *Science* **301**, 1503-1508 (2003). <https://doi.org:10.1126/science.1087025>

15 Howick, V. M. *et al.* The Malaria Cell Atlas: single parasite transcriptomes across the complete *Plasmodium* life cycle. *Science* **365**, eaaw2619 (2019). <https://doi.org:10.1126/science.aaw2619>

16 MalariaGen *et al.* Pf7: an open dataset of P*lasmodium falciparum* genome variation in 20,000 worldwide samples. *Wellcome Open Res* **8**, 22 (2023). <https://doi.org:10.12688/wellcomeopenres.18681.1>

17 Babai, R., Izrael, R. & Vertessy, B. G. Characterization of the dynamics of *Plasmodium falciparum* deoxynucleotide-triphosphate pool in a stage-specific manner. *Sci Rep* **12**, 19926 (2022). <https://doi.org:10.1038/s41598-022-23807-4>

18 Eaazhisai, K. *et al.* Crystal structure of fully ligated adenylosuccinate synthetase from *Plasmodium falciparum*. *J Mol Biol* **335**, 1251-1264 (2004). <https://doi.org:10.1016/j.jmb.2003.11.036>

19 Rossomando, E. F., Maldonado, B. & Crean, E. V. Effect of hadacidin on growth and adenylosuccinate synthetase activity of *Dictyostelium discoideum*. *Antimicrob Agents Chemother* **14**, 476-482 (1978). <https://doi.org:10.1128/AAC.14.3.476>

20 Raman, J., Mehrotra, S., Anand, R. P. & Balaram, H. Unique kinetic mechanism of *Plasmodium falciparum* adenylosuccinate synthetase. *Mol Biochem Parasitol* **138**, 1-8 (2004). <https://doi.org:10.1016/j.molbiopara.2004.06.013>

21 Wojtys, M. I. *et al.* A comprehensive method for determining cellular uptake of purine nucleoside phosphorylase and adenylosuccinate synthetase inhibitors by *H. pylori*. *Appl Microbiol Biotechnol* **105**, 7949-7967 (2021). <https://doi.org:10.1007/s00253-021-11510-9>

22 Bubic, A. *et al.* In the quest for new targets for pathogen eradication: the adenylosuccinate synthetase from the bacterium *Helicobacter pylori*. *J Enzyme Inhib Med Chem* **33**, 1405-1414 (2018). <https://doi.org:10.1080/14756366.2018.1506773>

23 Wang, C., Kruger, A., Du, X., Wrenger, C. & Groves, M. R. Novel highlight in malarial drug discovery: aspartate transcarbamoylase. *Front Cell Infect Microbiol* **12**, 841833 (2022). <https://doi.org:10.3389/fcimb.2022.841833>

24 Owoloye, A., Enejoh, O. A., Akanbi, O. M. & Bankole, O. M. Molecular docking analysis of *Plasmodium falciparum* dihydroorotate dehydrogenase towards the design of effective inhibitors. *Bioinformation* **16**, 672-678 (2020). <https://doi.org:10.6026/97320630016672>

25 Wang, C. *et al.* Discovery of small-molecule allosteric inhibitors of *Pf*ATC as antimalarials. *J Am Chem Soc* **144**, 19070-19077 (2022). <https://doi.org:10.1021/jacs.2c08128>

26 Lunev, S., Bosch, S. S., Batista Fde, A., Wrenger, C. & Groves, M. R. Crystal structure of truncated aspartate transcarbamoylase from *Plasmodium falciparum*. *Acta Crystallogr F Struct Biol Commun* **72**, 523-533 (2016). <https://doi.org:10.1107/S2053230X16008475>

27 Lunev, S. *et al.* Identification of a non-competitive inhibitor of *Plasmodium falciparum* aspartate transcarbamoylase. *Biochem Biophys Res Commun* **497**, 835-842 (2018). <https://doi.org:10.1016/j.bbrc.2018.02.112>

28 Singh, A. K. *et al.* Structural insights into acetylated histone ligand recognition by the BDP1 bromodomain of *Plasmodium falciparum*. *Int J Biol Macromol* **223**, 316-326 (2022). <https://doi.org:10.1016/j.ijbiomac.2022.10.247>

29 Josling, G. A. *et al.* A *Plasmodium Falciparum* bromodomain protein regulates invasion gene expression. *Cell Host Microbe* **17**, 741-751 (2015). <https://doi.org:10.1016/j.chom.2015.05.009>

30 Gouge, J. *et al.* Molecular mechanisms of Bdp1 in TFIIIB assembly and RNA polymerase III transcription initiation. *Nat Commun* **8**, 130 (2017). <https://doi.org:10.1038/s41467-017-00126-1>

31 Chua, M. J., Robaa, D., Skinner-Adams, T. S., Sippl, W. & Andrews, K. T. Activity of bromodomain protein inhibitors/binders against asexual-stage *Plasmodium falciparum* parasites. *Int J Parasitol Drugs Drug Resist* **8**, 189-193 (2018). <https://doi.org:10.1016/j.ijpddr.2018.03.001>

32 Hitz, E. *et al.* The catalytic subunit of *Plasmodium falciparum* casein kinase 2 is essential for gametocytogenesis. *Commun Biol* **4**, 336 (2021). <https://doi.org:10.1038/s42003-021-01873-0>

33 Ruiz-Carrillo, D. *et al.* The protein kinase CK2 catalytic domain from *Plasmodium falciparum*: crystal structure, tyrosine kinase activity and inhibition. *Sci Rep* **8**, 7365 (2018). <https://doi.org:10.1038/s41598-018-25738-5>

34 Grygier, P. *et al.* Silmitasertib (CX-4945), a clinically used CK2-Kinase inhibitor with additional effects on GSK3beta and DYRK1A kinases: a structural perspective. *J Med Chem* **66**, 4009-4024 (2023). <https://doi.org:10.1021/acs.jmedchem.2c01887>

35 AhYoung, A. P., Koehl, A., Vizcarra, C. L., Cascio, D. & Egea, P. F. Structure of a putative ClpS N-end rule adaptor protein from the malaria pathogen *Plasmodium falciparum*. *Protein Sci* **25**, 689-701 (2016). <https://doi.org:10.1002/pro.2868>

36 Mundra, S. *et al.* A novel class of Plasmodial ClpP protease inhibitors as potential antimalarial agents. *Bioorg Med Chem* **25**, 5662-5677 (2017). <https://doi.org:10.1016/j.bmc.2017.08.049>

37 Rathore, S. *et al.* A cyanobacterial serine protease of *Plasmodium falciparum* is targeted to the apicoplast and plays an important role in its growth and development. *Mol Microbiol* **77**, 873-890 (2010). <https://doi.org:10.1111/j.1365-2958.2010.07251.x>

38 Jain, S. *et al.* The prokaryotic ClpQ protease plays a key role in growth and development of mitochondria in *Plasmodium falciparum*. *Cell Microbiol* **15**, 1660-1673 (2013). <https://doi.org:10.1111/cmi.12142>

39 Rathore, S. *et al.* Disruption of a mitochondrial protease machinery in *Plasmodium falciparum* is an intrinsic signal for parasite cell death. *Cell Death Dis* **2**, e231 (2011). <https://doi.org:10.1038/cddis.2011.118>

40 Ng, C. L., Fidock, D. A. & Bogyo, M. Protein degradation systems as antimalarial therapeutic targets. *Trends Parasitol* **33**, 731-743 (2017). <https://doi.org:10.1016/j.pt.2017.05.009>

41 Pirovich, D. B., Da'dara, A. A. & Skelly, P. J. Multifunctional fructose 1,6-bisphosphate aldolase as a therapeutic target. *Front Mol Biosci* **8**, 719678 (2021). <https://doi.org:10.3389/fmolb.2021.719678>

42 Nemetski, S. M. *et al.* Inhibition by stabilization: targeting the *Plasmodium falciparum* aldolase-TRAP complex. *Malar J* **14**, 324 (2015). <https://doi.org:10.1186/s12936-015-0834-9>

43 Buscaglia, C. A., Coppens, I., Hol, W. G. & Nussenzweig, V. Sites of interaction between aldolase and thrombospondin-related anonymous protein in plasmodium. *Mol Biol Cell* **14**, 4947-4957 (2003). <https://doi.org:10.1091/mbc.e03-06-0355>

44 Thommen, B. T. *et al.* Genetic validation of *Pf*FKBP35 as an antimalarial drug target. *Elife* **12**, RP86975 (2023). <https://doi.org:10.7554/eLife.86975>

45 Bianchin, A., Allemand, F., Bell, A., Chubb, A. J. & Guichou, J. F. Two crystal structures of the FK506-binding domain of *Plasmodium falciparum* FKBP35 in complex with rapamycin at high resolution. *Acta Crystallogr D Biol Crystallogr* **71**, 1319-1327 (2015). <https://doi.org:10.1107/S1399004715006239>

46 Harikishore, A., Niang, M., Rajan, S., Preiser, P. R. & Yoon, H. S. Small molecule *Plasmodium* FKBP35 inhibitor as a potential antimalaria agent. *Sci Rep* **3**, 2501 (2013). <https://doi.org:10.1038/srep02501>

47 Milani, M. *et al.* Ferredoxin-NADP+ reductase from *Plasmodium falciparum* undergoes NADP+-dependent dimerization and inactivation: functional and crystallographic analysis. *J Mol Biol* **367**, 501-513 (2007). <https://doi.org:10.1016/j.jmb.2007.01.005>

48 Crobu, D. *et al.* *Plasmodium falciparum* ferredoxin-NADP+ reductase His286 plays a dual role in NADP(H) binding and catalysis. *Biochemistry* **48**, 9525-9533 (2009). <https://doi.org:10.1021/bi9013209>

49 Haeussler, K. *et al.* Glucose 6-phosphate dehydrogenase 6-phosphogluconolactonase: characterization of the *Plasmodium vivax* enzyme and inhibitor studies. *Malar J* **18**, 22 (2019). <https://doi.org:10.1186/s12936-019-2651-z>

50 Allen, S. M. *et al.* *Plasmodium falciparum* glucose-6-phosphate dehydrogenase 6-phosphogluconolactonase is a potential drug target. *FEBS J* **282**, 3808-3823 (2015). <https://doi.org:10.1111/febs.13380>

51 Jortzik, E. *et al.* Glucose-6-phosphate dehydrogenase-6-phosphogluconolactonase: a unique bifunctional enzyme from *Plasmodium falciparum*. *Biochem J* **436**, 641-650 (2011). <https://doi.org:10.1042/BJ20110170>

52 Berneburg, I. *et al.* An optimized dihydrodibenzothiazepine lead compound (SBI-0797750) as a potent and selective inhibitor of *Plasmodium falciparum* and *P. vivax* Glucose 6-Phosphate Dehydrogenase 6-Phosphogluconolactonase. *Antimicrob Agents Chemother* **66**, e0210921 (2022). <https://doi.org:10.1128/aac.02109-21>

53 Preuss, J. *et al.* Discovery of a *Plasmodium falciparum* glucose-6-phosphate dehydrogenase 6-phosphogluconolactonase inhibitor (R,Z)-N-((1-ethylpyrrolidin-2-yl)methyl)-2-(2-fluorobenzylidene)-3-oxo-3,4-dihydro-2H-benzo[b][1,4]thiazine-6-carboxamide (ML276) that reduces parasite growth in vitro. *J Med Chem* **55**, 7262-7272 (2012). <https://doi.org:10.1021/jm300833h>

54 Cova, M. *et al.* The Apicomplexa-specific glucosamine-6-phosphate N-acetyltransferase gene family encodes a key enzyme for glycoconjugate synthesis with potential as therapeutic target. *Sci Rep* **8**, 4005 (2018). <https://doi.org:10.1038/s41598-018-22441-3>

55 Chi, J. *et al.* Plasmodium falciparum Apicomplexan-Specific Glucosamine-6-Phosphate N-Acetyltransferase Is Key for Amino Sugar Metabolism and Asexual Blood Stage Development. *mBio* **11** (2020). <https://doi.org:10.1128/mBio.02045-20>

56 Dar, M. A., Sharma, A., Mondal, N. & Dhar, S. K. Molecular cloning of apicoplast-targeted Plasmodium falciparum DNA gyrase genes: unique intrinsic ATPase activity and ATP-independent dimerization of *Pf*GyrB subunit. *Eukaryot Cell* **6**, 398-412 (2007). <https://doi.org:10.1128/EC.00357-06>

57 Singh, P., Rani, K., Gotmare, A. & Bhattacharyya, S. A tale of topoisomerases and the knotty genetic material in the backdrop of Plasmodium biology. *Biosci Rep* **42** (2022). <https://doi.org:10.1042/BSR20212847>

58 Dias, M., Chapagain, T. & Leng, F. A fluorescence-based, T5 exonuclease-amplified DNA cleavage assay for discovering bacterial DNA gyrase poisons. *bioRxiv* (2023). <https://doi.org:10.1101/2023.10.16.562555>

59 Pakosz, Z., Lin, T. Y., Michalczyk, E., Nagano, S. & Heddle, J. G. Inhibitory compounds targeting *Plasmodium falciparum* gyrase B. *Antimicrob Agents Chemother* **65**, e0026721 (2021). <https://doi.org:10.1128/AAC.00267-21>

60 Tang Girdwood, S. C., Nenortas, E. & Shapiro, T. A. Targeting the gyrase of *Plasmodium falciparum* with topoisomerase poisons. *Biochem Pharmacol* **95**, 227-237 (2015). <https://doi.org:10.1016/j.bcp.2015.03.018>

61 Vanden Broeck, A., Lotz, C., Ortiz, J. & Lamour, V. Cryo-EM structure of the complete *E. coli* DNA gyrase nucleoprotein complex. *Nat Commun* **10**, 4935 (2019). <https://doi.org:10.1038/s41467-019-12914-y>

62 Vanden Broeck, A. *et al.* Structural basis for allosteric regulation of Human Topoisomerase IIalpha. *Nat Commun* **12**, 2962 (2021). <https://doi.org:10.1038/s41467-021-23136-6>

63 Y, V. T. M. *et al.* Inhibition and mechanism of *Plasmodium falciparum* hypoxanthine-guanine-xanthine phosphoribosyltransferase. *ACS Chem Biol* **17**, 3407-3419 (2022). <https://doi.org:10.1021/acschembio.2c00546>

64 Klejch, T. *et al.* Stereo-defined acyclic nucleoside phosphonates are selective and potent inhibitors of parasite 6-Oxopurine phosphoribosyltransferases. *J Med Chem* **65**, 4030-4057 (2022). <https://doi.org:10.1021/acs.jmedchem.1c01881>

65 Hazleton, K. Z. *et al.* Acyclic immucillin phosphonates: second-generation inhibitors of *Plasmodium falciparum* hypoxanthine-guanine-xanthine phosphoribosyltransferase. *Chem Biol* **19**, 721-730 (2012). <https://doi.org:10.1016/j.chembiol.2012.04.012>

66 Davis, M. I. *et al.* Identification of novel *Plasmodium falciparum* hexokinase inhibitors with antiparasitic activity. *Antimicrob Agents Chemother* **60**, 6023-6033 (2016). <https://doi.org:10.1128/AAC.00914-16>

67 Dillenberger, M. *et al.* Structural analysis of *Plasmodium falciparum* hexokinase provides novel information about catalysis due to a *Plasmodium*-specific insertion. *Int J Mol Sci* **24** (2023). <https://doi.org:10.3390/ijms241612739>

68 O'Rourke, P. E., Kalinowska-Tluscik, J., Fyfe, P. K., Dawson, A. & Hunter, W. N. Crystal structures of IspF from *Plasmodium falciparum* and *Burkholderia cenocepacia*: comparisons inform antimicrobial drug target assessment. *BMC Struct Biol* **14**, 1 (2014). <https://doi.org:10.1186/1472-6807-14-1>

69 Crane, C. M. *et al.* Fluorescent inhibitors for IspF, an enzyme in the non-mevalonate pathway for isoprenoid biosynthesis and a potential target for antimalarial therapy. *Angew Chem Int Ed Engl* **45**, 1069-1074 (2006). <https://doi.org:10.1002/anie.200503003>

70 Geist, J. G. *et al.* Thiazolopyrimidine inhibitors of 2-methylerythritol 2,4-cyclodiphosphate synthase (IspF) from *Mycobacterium tuberculosis* and *Plasmodium falciparum*. *ChemMedChem* **5**, 1092-1101 (2010). <https://doi.org:10.1002/cmdc.201000083>

71 Stack, C. M. *et al.* Characterization of the *Plasmodium falciparum* M17 leucyl aminopeptidase. A protease involved in amino acid regulation with potential for antimalarial drug development. *J Biol Chem* **282**, 2069-2080 (2007). <https://doi.org:10.1074/jbc.M609251200>

72 Mistry, S. N. *et al.* Two-pronged attack: dual inhibition of *Plasmodium falciparum* M1 and M17 metalloaminopeptidases by a novel series of hydroxamic acid-based inhibitors. *J Med Chem* **57**, 9168-9183 (2014). <https://doi.org:10.1021/jm501323a>

73 Izquierdo, M. *et al.* Identification of a potent and selective LAPTc inhibitor by RapidFire-Mass Spectrometry, with antichagasic activity. *PLoS Negl Trop Dis* **18**, e0011956 (2024). <https://doi.org:10.1371/journal.pntd.0011956>

74 Robien, M. A. *et al.* An improved crystal form of *Plasmodium falciparum* peptide deformylase. *Protein Sci* **13**, 1155-1163 (2004). <https://doi.org:10.1110/ps.03456404>

75 Silver, L. L. Challenges of antibacterial discovery. *Clin Microbiol Rev* **24**, 71-109 (2011). <https://doi.org:10.1128/CMR.00030-10>

76 Kumar, A. *et al.* Crystals of peptide deformylase from *Plasmodium falciparum* reveal critical characteristics of the active site for drug design. *Structure* **10**, 357-367 (2002). <https://doi.org:10.1016/s0969-2126(02)00719-0>

77 Bracchi-Ricard, V. *et al.* Characterization of an eukaryotic peptide deformylase from *Plasmodium falciparum*. *Arch Biochem Biophys* **396**, 162-170 (2001). <https://doi.org:10.1006/abbi.2001.2631>

78 Goodman, C. D., Uddin, T., Spillman, N. J. & McFadden, G. I. A single point mutation in the *Plasmodium falciparum* FtsH1 metalloprotease confers actinonin resistance. *Elife* **9** (2020). <https://doi.org:10.7554/eLife.58629>

79 Tehlan, A., Bhowmick, K., Kumar, A., Subbarao, N. & Dhar, S. K. The tetrameric structure of *Plasmodium falciparum* phosphoglycerate mutase is critical for optimal enzymatic activity. *J Biol Chem* **298**, 101713 (2022). <https://doi.org:10.1016/j.jbc.2022.101713>

80 Huang, K. *et al.* A novel Allosteric inhibitor of phosphoglycerate mutase 1 suppresses growth and metastasis of non-small-cell lung cancer. *Cell Metab* **30**, 1107-1119 e1108 (2019). <https://doi.org:10.1016/j.cmet.2019.09.014>

81 Pandey, R., Gupta, P., Mohmmed, A., Malhotra, P. & Gupta, D. A *Plasmodium falciparum* protein tyrosine phosphatase inhibitor identified from the ChEMBL-NTD database blocks parasite growth. *FEBS Open Bio* **11**, 1921-1929 (2021). <https://doi.org:10.1002/2211-5463.13171>

82 Gulerez, I. *et al.* Phosphocysteine in the PRL-CNNM pathway mediates magnesium homeostasis. *EMBO Rep* **17**, 1890-1900 (2016). <https://doi.org:10.15252/embr.201643393>

83 Van Wye, J. *et al.* Identification and localization of rab6, separation of rab6 from ERD2 and implications for an 'unstacked' Golgi, in *Plasmodium falciparum*. *Mol Biochem Parasitol* **83**, 107-120 (1996). <https://doi.org:10.1016/s0166-6851(96)02759-4>

84 Achila, D., Gulati, M., Jain, N. & Britton, R. A. Biochemical characterization of ribosome assembly GTPase RbgA in *Bacillus subtilis*. *J Biol Chem* **287**, 8417-8423 (2012). <https://doi.org:10.1074/jbc.M111.331322>

85 Pausch, P. *et al.* Structural basis for (p)ppGpp-mediated inhibition of the GTPase RbgA. *J Biol Chem* **293**, 19699-19709 (2018). <https://doi.org:10.1074/jbc.RA118.003070>

86 Sopitthummakhun, K. *et al.* *Plasmodium* serine hydroxymethyltransferase as a potential anti-malarial target: inhibition studies using improved methods for enzyme production and assay. *Malar J* **11**, 194 (2012). <https://doi.org:10.1186/1475-2875-11-194>

87 Nonaka, H. *et al.* Design strategy for serine hydroxymethyltransferase probes based on retro-aldol-type reaction. *Nat Commun* **10**, 876 (2019). <https://doi.org:10.1038/s41467-019-08833-7>

88 Witschel, M. C. *et al.* Inhibitors of plasmodial serine hydroxymethyltransferase (SHMT): cocrystal structures of pyrazolopyrans with potent blood- and liver-stage activities. *J Med Chem* **58**, 3117-3130 (2015). <https://doi.org:10.1021/jm501987h>

89 Kandeel, M. & Kitade, Y. Molecular characterization, heterologous expression and kinetic analysis of recombinant *Plasmodium falciparum* thymidylate kinase. *J Biochem* **144**, 245-250 (2008). <https://doi.org:10.1093/jb/mvn062>

90 Cassera, M. B., Zhang, Y., Hazleton, K. Z. & Schramm, V. L. Purine and pyrimidine pathways as targets in *Plasmodium falciparum*. *Curr Top Med Chem* **11**, 2103-2115 (2011). <https://doi.org:10.2174/156802611796575948>

91 Chen, H. *et al.* Crystal structure of the extracellular domain of the receptor-like kinase TMK3 from *Arabidopsis thaliana*. *Acta Crystallogr F Struct Biol Commun* **76**, 384-390 (2020). <https://doi.org:10.1107/S2053230X20010122>

92 Dar, A., Godara, P., Prusty, D. & Bashir, M. *Plasmodium falciparum* topoisomerases: emerging targets for anti-malarial therapy. *Eur J Med Chem* **265**, 116056 (2024). <https://doi.org:10.1016/j.ejmech.2023.116056>

93 Juul, S. *et al.* Droplet microfluidics platform for highly sensitive and quantitative detection of malaria-causing *Plasmodium* parasites based on enzyme activity measurement. *ACS Nano* **6**, 10676-10683 (2012). <https://doi.org:10.1021/nn3038594>

94 Gu, M. *et al.* Fluorescently labeled circular DNA molecules for DNA topology and topoisomerases. *Sci Rep* **6**, 36006 (2016). <https://doi.org:10.1038/srep36006>

95 Bodley, A. L., Cumming, J. N. & Shapiro, T. A. Effects of camptothecin, a topoisomerase I inhibitor, on *Plasmodium falciparum*. *Biochem Pharmacol* **55**, 709-711 (1998). <https://doi.org:10.1016/s0006-2952(97)00556-x>

96 Cortopassi, W. A. *et al.* Theoretical and experimental studies of new modified isoflavonoids as potential inhibitors of topoisomerase I from *Plasmodium falciparum*. *PLoS One* **9**, e91191 (2014). <https://doi.org:10.1371/journal.pone.0091191>

97 Frickel, E. M. *et al.* Apicomplexan UCHL3 retains dual specificity for ubiquitin and Nedd8 throughout evolution. *Cell Microbiol* **9**, 1601-1610 (2007). <https://doi.org:10.1111/j.1462-5822.2007.00896.x>

98 Artavanis-Tsakonas, K. *et al.* Characterization and structural studies of the *Plasmodium falciparum* ubiquitin and Nedd8 hydrolase UCHL3. *J Biol Chem* **285**, 6857-6866 (2010). <https://doi.org:10.1074/jbc.M109.072405>

99 Imhoff, R. D. *et al.* Identification of covalent fragment inhibitors for *Plasmodium falciparum* UCHL3 with anti-malarial efficacy. *Bioorg Med Chem Lett* **94**, 129458 (2023). <https://doi.org:10.1016/j.bmcl.2023.129458>

100 Miles, A. *et al.* Indels, structural variation, and recombination drive genomic diversity in *Plasmodium falciparum*. *Genome Res* **26**, 1288-1299 (2016). <https://doi.org:10.1101/gr.203711.115>

101 Sayers, E. W. *et al.* Database resources of the national center for biotechnology information. *Nucleic Acids Res* **50**, D20-D26 (2022). <https://doi.org:10.1093/nar/gkab1112>

102 Berman, H. M. *et al.* The Protein Data Bank. *Nucleic Acids Res* **28**, 235-242 (2000). <https://doi.org:10.1093/nar/28.1.235>
